# Achieving therapeutic antibiotic levels during intermittent dosing of meropenem and piperacillin-tazobactam in critically ill children: the ATACC study

**DOI:** 10.1128/aac.01968-25

**Published:** 2026-05-28

**Authors:** Ari R. Joffe, Ashley Humber, Angela Bates, Jeffrey Lipman, Steven Wallis, Jason Roberts

**Affiliations:** 1 Department of Pediatrics, Division of Critical Care Medicine, University of Alberta 3158, Edmonton, Alberta, Canada; 2 Department of Pediatrics, University of Calgary 2129, Calgary, Alberta, Canada; 3 University of Queensland Centre for Clinical Research, Faculty of Health, Medicine and Behavioural Sciences, The University of Queensland 1974, Brisbane, Australia; 4 Herston Infectious Diseases Institute, Metro North Health, Brisbane, Australia; 5 Department of Pharmacy, Royal Brisbane and Women’s Hospital, Brisbane, Australia; 6 Department of Intensive Care Medicine, Royal Brisbane and Women’s Hospital, Brisbane, Australia; 7 Division of Anesthesia Critical Care and Emergency and Pain Medicine, University of Montpellier, Nimes University Hospital, Nimes, France; University Children's Hospital Münster, Münster, Germany

**Keywords:** beta-lactam, critical care, meropenem, pediatrics, pharmacodynamics, piperacillin-tazobactam, therapeutic drug monitoring

## Abstract

Achieving antibiotic pharmacodynamic targets is critical to surviving sepsis. The purpose of this study was to determine the achievement of pharmacodynamic target—free minimum plasma concentration (fCmin)—during the first 2 days of high-dose 6-h intravenous meropenem or piperacillin-tazobactam in critically ill children, and to identify its predictors. This was a prospective observational study conducted in two pediatric intensive care units of children admitted between 2022 and 2023 who were prescribed meropenem or piperacillin-tazobactam. Primary outcomes were achievement of pharmacodynamic target fCmin (defined as fCmin>MIC or fCmin>4×MIC). Secondary outcomes included predictors of target concentrations and time to resolution of signs of severe infection using univariate and multivariable regressions. Of 49 patients, 47 had an antibiotic concentration measured at 24 h, and 38 at 48 h. At 24 h, 48 h, or either time point, augmented renal clearance (ARC) occurred in 13/30 (43%), 14/22 (64%), and 17/30 (57%), respectively. For epidemiologic cutoff values, fCmin>MIC occurred in 17/47 (36%) and 13/38 (34%) at 24 and 48 h, and fCmin>4×MIC occurred in 3/47 (6%) and 4/38 (11%). Target tazobactam concentrations (fCmin>0.5 or >2mg/L) occurred in 48% or 64% of the patients and 28% or 39% of the patients at 24 and 48 h. Potentially neurotoxic concentrations occurred in 1/9 (11%) patients on meropenem at 48 h, and in none receiving piperacillin-tazobactam. Only ARC independently predicted not achieving fCmin>MIC at 48 h (odds ratio 0.04 [95% CI 0.00, 0.69] *P* = 0.026). Therapeutic antibiotic concentration did not predict the time to resolution of severe symptoms and signs of sepsis (*n* = 32). In this cohort of critically ill children, high doses of piperacillin-tazobactam or meropenem did not often achieve pharmacodynamic targets and were rarely toxic.

## INTRODUCTION

In sepsis and septic shock, early and appropriate antibiotics are a crucial component in the chain of survival (1). Appropriate antibiotic therapy refers to not only the right spectrum of activity but also the right dosing in order to achieve therapeutic antibiotic concentrations at the site of infection (2, 3). Pharmacodynamics describes the relationship between drug concentration and effect and can be used to define an antibiotic target concentration that best achieves bacterial eradication (4, 5). For beta-lactam antibiotics, this target for maximal bactericidal activity and clinical cure is the fraction of time that the free antibiotic concentration at the site of infection is above a multiple of the minimal inhibitory concentration (MIC) of the bacterial organism, with recommendations of 100% fT>MIC or fT>4×MIC (4, 5). In this study, the plasma trough concentration was the minimum concentration (Cmin), indicating the concentration for 100% of the dosing interval; therefore, to indicate 100% fT above a multiple of MIC, the notation fCmin>MIC or fCmin>4×MIC was used.

There are many reasons why current beta-lactam antibiotic dosing may not achieve target concentrations in critically ill patients, and why this problem is particularly important in critically ill patients (Table 1) (2–21). For these reasons, blood and tissue concentrations of antibiotics in critically ill patients have often failed to achieve pharmacodynamic targets (4, 5, 9). In children, studies have been limited by frequent use of convenience sampling to extrapolate the fCmin (9, 22, 23), variable doses of beta-lactams not optimized for pharmacodynamics (22–26), and variable definitions of ARC (6, 8, 27).

**TABLE 1:** Reasons why current beta-lactam antibiotic dosing may not achieve target concentrations in critically ill children

Reason	Details
Changes in pharmacokinetics in critically ill children	Augmented renal clearance (ARC) due to recruitment of underlying renal functional reserve (i.e., increased glomerular filtration rate) by the hyperdynamic vasodilatory circulation with increased cardiac output often supported with volume resuscitation and vasoactive infusions (2, 4–8).
	Increased volume of distribution (Vd) due to fluid therapy, capillary leak, and hypoalbuminemia (3–5, 9). These can be exacerbated by therapies including renal replacement therapy (RRT) and extracorporeal membrane oxygenation (ECMO) (2). Some population meropenem PK modeling suggested that fluid overload (which contributes to increased central Vd) results in a lower drug concentration in the central Vd, in turn, resulting in a longer elimination half-life, and possibly better Cmin (10, 11). This may not apply on the first day of therapy prior to pharmacokinetic steady state. In addition, given the poor tissue penetration of beta-lactams in the critically ill, tissue compartment fluid overload may dilute the achieved concentration at the site of infection (12–16).
Changes in pharmacodynamics in critically ill children	Extrapolation from measured plasma concentrations results in an overestimation of extracellular tissue interstitial fluid antibiotic penetration at the site of infection (12–16). Antibiotic concentrations in tissues for piperacillin and meropenem, including (at least) in subcutaneous, muscle, and lung epithelial lining fluid, determined using microdialysis, are often less than half those in blood due to low tissue penetration ratios (12–16).
Subtherapeutic concentrations are potentially of even more concern due to changes in pathogen clearance in critically ill children	Critically ill patients are often infected with organisms that have high MIC (e.g., multidrug-resistant bacteria) (2, 3, 17). In addition, the measured MIC is only accurate within one to two dilutions, meaning that the MIC could be at least two times higher than that reported (4, 5, 18).
	Critically ill patients have altered immune function, increasing the reliance on bactericidal antibiotic tissue concentrations to eradicate an infection (19, 20).
	Critically ill patients can be reservoirs for the development of antibiotic resistance, the prevention of which may require concentrations exceeding those associated with clinical efficacy (21).

We designed this prospective observational study to fill gaps in the pediatric literature. We aimed to determine the achievement of therapeutic plasma fCmin during the first 2 days of high doses of meropenem or piperacillin-tazobactam antibiotic treatment in critically ill children and to determine the predictors of achieving target fCmin. Based on the reasons given in Table 1, and the previous studies (despite their limitations) of failure to achieve pharmacodynamic targets, we hypothesized that failure to achieve the pharmacodynamic target fCmin would occur in over 50% of patients and that ARC would be a predictor of failure to achieve the pharmacodynamic target.

## MATERIALS AND METHODS

The study was approved by the health research ethics board of the University of Alberta (study ID Pro00104611, expiry October 1, 2025), and all guardians signed an informed consent form for participation. The research was conducted in accordance with the Declaration of Helsinki and national and institutional standards.

This prospective observational study was conducted in two pediatric intensive care units (PICU) at one referral children’s hospital. Eligibility criteria included age between 1 month and 17 years, admitted between Jan 2022 and May 2023, prescribed a high dose (according to institutional suggestions) of meropenem or piperacillin-tazobactam intravenously by the clinical team, and anticipated duration of the antibiotic for at least 48 h. Patients with acute (AKI) or chronic kidney injury sufficient to require adjusted renal dosing of the antibiotic according to the clinical team’s judgment, expected not to survive for 48 h, or lacking bloodwork access (i.e., arterial line, central venous line, or scheduled peripheral labwork at the appropriate time) were excluded. Screening occurred during weekdays by the clinical pharmacist for patients prescribed meropenem or piperacillin-tazobactam, and the guardian was asked for consent to be fully screened and approached for signed informed consent by the research coordinator if eligible.

Included patients were those prescribed a high dose of meropenem (120 mg/kg/day, divided q6h to a maximum of 1.5 g q6h) or piperacillin-tazobactam (300 mg/kg/day, divided q6h to a maximum of 4.5 g [4 g of piperacillin] q6h), each dose administered over 30 min. Although certain sources do recommend maximal doses of piperacillin of 400 mg/kg/day, our institution does not allow doses above 300 mg/kg/day (28).

The following data were recorded from patient charts: demographics, including age, sex, body surface area (BSA), and admission diagnostic category; infection details, including suspected infection site, confirmed infection (defined as a clinical team decision to treat with antibiotics for ≥5 days, and final blinded adjudication by two pediatric infectious diseases specialists), positive cultures with the organism(s) and their MIC (provided at the end of the study, as the clinical laboratory does not routinely report these data); and severity of illness details at the time of antibiotic order, including pediatric logistic organ dysfunction score (PELOD-2) (29), vasoactive inotrope score (30), acute respiratory distress syndrome (ARDS) category (mild, moderate, and severe) (31), AKI category (32), sepsis category (sepsis, severe sepsis, and septic shock) (33), ventilation (invasive, non-invasive, and high-flow nasal cannula), % of fluid accumulation since PICU admission (or in the past 72 h if admission had been for >5 days), continuous RRT, and ECMO. Outcomes determined were hospital mortality by 30 days from antibiotic prescription and PICU time to resolution of signs of severe infection (defined as final blinded adjudication by two infectious diseases specialists, based upon their judgment that vasoactive inotrope score, ventilator settings, level of consciousness, and temperature had returned to baseline or a new baseline after the intercurrent sepsis episode).

A case report form and its manual, finalized prior to the start of the study, provided precise definitions of these variables (Supplemental forms).

To determine creatinine clearance (CrCl) in those with an indwelling urinary catheter, a urine sample was collected over 4–6 h as close to the 24 and 48 h time points as possible, and CrCl was calculated and normalized to 1.73 m² BSA. In adults, a CrCl>130 mL/min/1.73 m² (2) (i.e., ≥90th percentile of population norms) is considered ARC (34); therefore, CrCl ≥90th percentile for age was used to define ARC for this patient population (35, 36). A sensitivity analysis using a CrCl ≥75th percentile found in this study aimed to explore an alternative definition of ARC. Definitions of 90th percentile of CrCl by age are given in the case report form manual (Supplemental forms). For those patients without a measured CrCl, estimated GFR based on formulas was not determined, as these were considered inaccurate in critically ill patients (37, 38).

Plasma antibiotic concentration was measured from 1.5 mL of blood at the 24 and 48 h time points on antibiotic therapy and sent to an independent third-party laboratory for batch analysis. The blood sample was collected in a lithium heparin tube, placed on ice, transported to our university research laboratory, where it was centrifuged for 10 min at 2,000 × *g* (within 6 h of collection), and 50 µL of plasma was transferred into a labeled (with the patient study ID number) cryovial. The cryovials were stored at −70°C and shipped frozen with dry ice in batches to the University Center for Clinical Research (UQCCR), the University of Queensland, Brisbane, Australia. Bioanalysis was performed using ultra-high performance liquid chromatography–tandem mass spectrometry, as described previously (39, 40). The measurement was for total plasma antibiotic concentration (for meropenem, piperacillin, and tazobactam), and this was corrected to free concentration for piperacillin-tazobactam, assuming 20% protein binding; no correction was used for meropenem, as there is only 0%–2% protein binding (5). Clinicians and research coordinators recording data were blinded to antibiotic concentration measurements, and antibiotic measurements were performed blinded to clinical information.

The primary outcomes were frequency of achievement of the two target antibiotic concentrations, defined as fCmin>MIC and fCmin>4×MIC (4, 5). When a pathogen was cultured, the clinical laboratory MIC was determined using VITEK-2 (bioMérieux system for automated identification and antimicrobial susceptibility testing), and if indicated, confirmed with other methods (disk diffusion, broth microdilution, or E-tests), according to the Clinical and Laboratory Standards Institute (CLSI) guidelines. The European Committee on Antimicrobial Susceptibility Testing (EUCAST) epidemiological cutoff value (ECOFF) MIC, which indicated the highest MIC for *Pseudomonas aeruginosa* wild-type strains devoid of phenotypically detectable acquired resistance mechanisms, was also used (4, 5). The ECOFF values for *P. aeruginosa* were chosen because this is recommended for empiric therapy (aimed to cover the maximum of usual bacteria causing infection) (5), and *P. aeruginosa* is not an uncommon pathogen in PICU sepsis (e.g., 28% of GNB and 12% of isolated pathogens in a prospective international study) (41), especially with underlying chronic diseases (17, 42). Given the imprecision of clinical laboratory-measured MIC, which can be off by 1–2 dilutions, it is recommended to use the ECOFF when determining optimal pharmacodynamics of antibiotics, and therefore, the ECOFF MIC value was used for primary outcomes (4, 5, 18). The ECOFF MIC values used were 2 mg/L for meropenem and 16 mg/L for piperacillin. The target Cmin thresholds examined were fCmin>2 or 8 mg/L for meropenem, and fCmin 16 or 64 mg/L for piperacillin, that is, fCmin>ECOFF MIC or >4× ECOFF MIC. The incidence of potentially toxic concentrations of antibiotics was also determined, using concentration thresholds suggested by recent studies and reviews (4, 5, 43, 44). Toxicity risk for piperacillin-tazobactam was defined as piperacillin fCmin>160mg/L (although the literature was unclear whether this was for total or free concentration, to be conservative, we used free concentration) (4, 5), and for meropenem was defined as fCmin>44.5 (for nephrotoxicity) (4) or >64mg/L (for neurotoxicity) (5). Post-hoc, for any patient with a potentially toxic fCmin as defined above, a chart review was done for evidence of clinical nephrotoxicity (defined as worsening in measured urea or creatinine in the following 72 h) or neurotoxicity (defined as a change in sedation level or worsened level of consciousness, or seizures in the following 72 h). For tazobactam, the suggested concentration for efficacy was used, an fCmin>0.25 or 2 mg/L, based on a systematic review (45). Secondary outcomes included predictors of inadequate therapeutic antibiotic concentration, and in those with confirmed infection, time to resolution of signs of severe infection.

### Statistical analysis

The results are presented using mean (standard deviation, SD) or median (interquartile range, IQR) for continuous variables, and as counts (percentages) for categorical variables. For the primary outcome, the incidence of reaching therapeutic antibiotic fCmin concentrations of meropenem and piperacillin-tazobactam was determined and reported as target attainment percentages with adjusted Wald 95% confidence intervals (CI). The sample size aimed to have a narrow 95% CI for reaching pharmacodynamic target fCmin overall; with *n* = 50, and an incidence of 40%–50%, we estimated a reasonable adjusted Wald 95% CI of +/– 13%. For the secondary outcome, to determine the predictors of inadequate therapeutic antibiotic concentration, univariate logistic regression was used, and variables with *P* ≤ 0.10 were entered into multiple logistic regression, after ruling out multicollinearity. Variables pre-specified to be forced into the multiple logistic regression due to hypothesized clinical relevance were ARC and antibiotics used. For the secondary outcome, to determine the predictors of time to resolution of signs of severe infection, univariate linear regression was used, and variables with *P* ≤ 0.10 were entered into multiple linear regression, after ruling out multicollinearity. The same two clinical variables and fCmin>ECOFF MIC were pre-specified to be forced into the multiple linear regression. Post-hoc, predictors of ARC were examined using univariate and multiple logistic regression as described above. Each multiple regression had up to three variables, aiming to consider only one predictor per 5–10 events. In all multiple regressions, a *P*-value ≤ 0.05 was considered statistically significant. Analyses were done using SPSS v30.

This study was done and reported in accordance with the STROBE guidelines (see checklist in Supplemental material 2).

## RESULTS

### Cohort description

**Fig 1:**
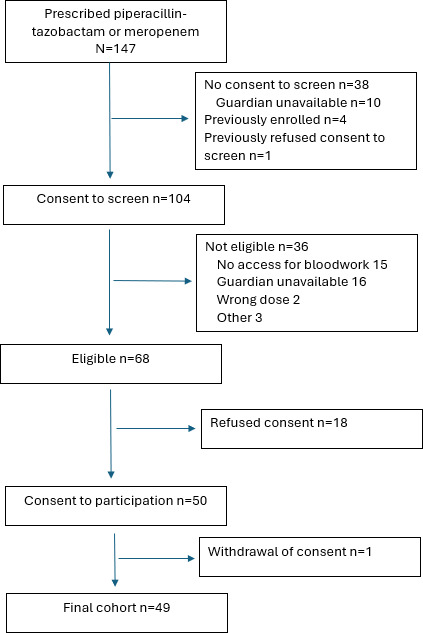
Patient inclusion in the ATACC study.

Of 147 patients prescribed piperacillin-tazobactam or meropenem, 104 consented to be screened, 68 (65%) of these were eligible, 50 (74%) of these consented, and one withdrew consent, leaving *n* = 49 patients included in the study (Fig. 1). Details of demographics, infection information, and severity of illness are given in Table 2 (see also eTable 1 in Supplemental material 2).

**TABLE 2:** Descriptive data for the 49 critically ill children included in the study^*a*^^*,^b^,*^^*c*^

Variable	Result (*n* = 49)	
Demographics		
Age (mo)	58 (72); 17 [6, 116]	
Sex (female)	17 (35%)	
Weight (kg)	18.4 (20.1); 10.4 [5.7, 20.0]	
Height (cm)	122 (216)	
Body Surface Area (m^2^)	0.66 (0.52); 0.47 [0.30, 0.92]	
Admission diagnostic category(ies)		
Sepsis	5	
Neurological	6	
Respiratory	11	
Cardiac	2	
Abdominal	1	
Renal	1	
Surgical cardiac	14	
Surgical non-cardiac	12	
Surgical transplant	6	
Infection information		
Infection type		
Community acquired	10 (20%)	
Nosocomial	39 (80%)	
Infection confirmation		
Treated ≥ 5 days with antibiotics	33 (67%)	
Treated < 5 days of antibiotics	16 (33%)	
Confirmed	34 (69%)	
Sepsis	47 (96%)	
Severe sepsis	47 (96%)	
Septic shock	18 (37%)	
Hypotension	11 (22%)	
Vasoactive infusion	14 (29%)	
Impaired perfusion	5 (10%)	
Site(s) of infection	34 (69%)	
Respiratory (pneumonia)	20	
Ventilator associated pneumonia	13	
Hospital acquired pneumonia	2	
Community acquired pneumonia	5	
Cardiac (mediastinitis)	1	
Abdominal	6	
Bowel perforation	1	
Intra-abdominal abscess	2	
Other (peritonitis, necrotizing, enterocolitis, ischemic bowel)	3	
Urinary Tract infection	7	
CLABSI (CVL, PICC, IVAD)	3	
Not confirmed or prophylaxis	15 (31%)	
Infecting organism		
Gram-negative bacilli	16 (33%)	
Gram-positive cocci	9 (18%)	
Gram-negative cocci/coccobacilli	4 (8%)	
No positive culture	25 (51%)	
Clinical condition at time of antibiotic prescription		
Variable at baseline or 24 or 48 h	**24 h**	**48 h**
Creatinine clearance	*n* = 30	*n* = 22
Serum creatinine (µmol/L)	37 (26)	34 (26)
Urine creatinine (µmol/L)	2,893 (3,168)	3,555 (5,170)
Urine volume (mL)	200 (196)	177 (178)
Minutes of urine collection (min)	266 (135)	245 (27)
Creatinine clearance (mL/min/1.73 m)	110 (81); 107 [57, 155]	118 (65); 132 [60, 169]
Augmented renal clearance (>90^th^ percentile for age)^*d*^	13/30 (43%)	14/22 (64%)
On RRT when level drawn	1	0
On ECMO, when the level was drawn	3	3
PELOD-2	5.1 (2.2); 5 [3, 6.5]	
Vasoactive inotrope score	3.6 (5.7); 0 [0, 5.5]	
On vasoactive(s)	22 (45%)	
Acute respiratory distress syndrome	24 (49%)	
Acute kidney injury	4 (8%)	
Urine output > 0.5 mL/kg/h	46 (94%)	
Ventilated	45 (92%)	
Invasive ventilation	38 (78%)	
Non-Invasive ventilation	7 (14%)	
Fluid accumulation since admission (%)	7.3 (10.2); 5.2 [0.9, 12.8]	
Fluid accumulation ≥ 10%	15 (31%)	
Outcomes		
Time to resolution of signs of severe infection (*n* = 34), h	84.3 (68.6); 65.0 [36.9, 111.0]; range 10-324	
Mortality within 30 days	2 (4%)	

^*a*^ Continuous variables are presented as mean (standard deviation); median [interquartile range].

^*b*^ Categorical variables are presented as number (percentage).

^*c*^ CLABSI, Central Line Associated Bloodstream Infection; CVL, central venous line; ECMO, extracorporeal membrane oxygenation; IVAD, implanted venous access device; PELOD-2, pediatric logistic organ dysfunction score; PICC, peripherally inserted central line; RRT, renal replacement therapy.

^*d*^ ARC was measured at both time points in n = 22: changed from yes to no, n = 0 (0%); and changed from no to yes, n = 4 (18%); both yes n = 10 (45%); and both no n = 8 (36%). ARC occurred at either time point in 17/30 (57%).

At 24 h, 48 h, or at either of the two time points on antibiotic therapy, ARC was documented in 13/30 (43%), 14/22 (64%), and 17/30 (57%) patients, respectively. The CrCl was measured at both time points in *n* = 22, and change from ARC to no-ARC did not occur; however, change from no-ARC to ARC occurred for four patients (18%).

### Primary outcome: antibiotic therapeutic concentration

Overall, 47 patients had an antibiotic concentration measured at 24 h and 38 at 48 h (Fig. S1 and Supplementary material 2). An infecting organism was known (and hence an MIC known) for *n* = 15 and *n* = 12, respectively. The proportion achieving fCmin>MIC for ECOFF values was 17/47 (36%; 95% CI 24%, 51%) and 13/38 (34%; 95% CI 21%, 50%) at 24 and 48 h, and the proportion achieving fCmin>4×MIC for ECOFF values was 3/47 (6%; 2%, 18%) and 4/38 (11%; 95% CI 4%, 25%) at 24 and 48 h. When MIC was directly measured and known, these proportions were higher; however, a majority still did not have fCmin>4×MIC (6/15 [40%] and 4/12 [33%]) at 24 and 48 h, respectively. Only one patient with a directly measured and known MIC had a resistant organism, with *Enterobacter cloacae* isolated from urine and a piperacillin MIC ≥128mg/L, and had a time to resolution of 123 h. Details of piperacillin-tazobactam and meropenem separately are provided in Table 3. When measured at both 24 and 48 h (*n* = 38), the fCmin>MIC by ECOFF values occurred in 11/38 (29%) at both times. The correlation between concentrations at 24 h and 48 h in the *n* = 38 patients was Pearson r = 0.46 (*P* = 0.003) (Fig. S2 and Supplementary material 2).

**TABLE 3:** Measured antibiotic concentrations, including therapeutic or toxic concentrations^*a*^

Variable	24 h	48 h
Piperacillin		
Number of patients	36	29
Antibiotic concentration	17.6 (21.7); 9.5 [2.5, 24.2]	16.5 (30.1); 3.1 [1.5, 13.0]
MIC known (mg/L)	10/36 (28%) (median 6.0, range 4.0, 128.0)	8/29 (28%) (median 6.0, range 4.0, 16.0)
fCmin > MIC	4/10 (40%)	3/8 (38%)
fCmin > 4 xMIC	2/10 (20%)	1/8 (13%)
fCmin > 16 mg/L (MIC)	10/36 (28%)	6/29 (21%)
fCmin > 64 mg/L (4x MIC)	1/36 (3%)	2/29 (7%)
Toxicity (fCmin > 160 mg/L) (8x MIC)	0/36 (0%)	0/29 (0%)
Meropenem		
Number of patients	11	9
Antibiotic Concentration	8.0 (15.1); 2.8 [1.1, 4.1]	16.8 (32.1); 2.6 [1.7, 21.2]
MIC known (mg/L)	5/11 (45%) (median 0.20, range 0.20, 0.20)	4/9 (44%) (median 0.20, range 0.20, 0.20)
fCmin > MIC	5/5 (100%)	4/4 (100%)
fCmin > 4 ×MIC	4/5 (80%)	3/4 (75%)
fCmin > 2 mg/L (MIC)	7/11 (64%)	7/9 (78%)
fCmin > 8 mg/L (4× MIC)	2/11 (18%)	2/9 (22%)
fCmin > 16 mg/L (8× MIC)	2/11 (18%)	2/9 (22%)
Toxicity (fCmin > 44.5 or 64 mg/L)	1/11 (9%) or 0 (0%)	1/9 (11%)
Combined		
Number of patients	47	38
MIC known	15	12
fCmin > MIC	9/15 (60%)	7/12 (58%)
fCmin > 4 ×MIC	6/15 (40%)	4/12 (33%)
fCmin > ECOFF MIC^*b*^	17/47 (36%)	13/38 (34%)
fCmin > ECOFF MIC×4	3/47 (6%)	4/38 (11%)
Toxicity^*c*^	0-1/47 (0-2%)	1/38 (3%)
Tazobactam		
Number of patients	36	29
Tazobactam concentration^*d*^	2.430 (2.858); 1.310 [0.500, 3.291]; 0 to 12.324	2.130 (3.653); 0.50 [0.50, 2.276]; 0 to 16.232
fCmin > 0.5 mg/L	23/36 (64%)	14/29 (48%)
fCmin > 2 mg/L	14/36 (39%)	8/29 (28%)

^*a*^ ECOFF, European Committee on Antimicrobial Susceptibility Testing (EUCAST) epidemiological cutoff value, which indicated the highest MIC for *P. aeruginosa *wild-type strains devoid of phenotypically detectable acquired resistance mechanisms; fCmin, 100% of time that the free antibiotic concentration was above a certain value (Cmin indicated the minimum concentration, at the end of the dosing interval, and therefore was the concentration that was achieved for 100% of the time); MIC, minimal inhibitory concentration.

^*b*^ Measured fCmin>ECOFF MIC, when done at both 24 h and 48 h (n = 38): changed from no to yes n = 2 (5%); changed from yes to no n = 3 (8%; 1 where first level was done too early); no at both times n = 22 (58%); yes at both times n = 11 (29%).

^*c*^ Toxicity in n=2: n=1 of these patients met criteria for acute kidney injury within 4 hours of meropenem prescription. Toxicity risk for piperacillin-tazobactam defined as when fCmin >160 mg/L (although unclear whether this was for total or free concentration; to be conservative, we used free concentration)(4, 5) and for meropenem when fCmin >44.5 (for nephrotoxicity)(4) or >64 mg/L (for neurotoxicity) (5).

^*d*^ When below the limit of quantitation (<0.625 mg/L) but not zero, a Cmin of 0.5 mg/L was imputed. Given the limit of detection, we chose 0.5 mg/L as the limit for efficacy for some pathogens (instead of 0.25 mg/L), and 2 mg/L as the limit for efficacy for some pathogens.

Tazobactam concentrations were also measured in *n* = 36 and *n* = 29 patients at 24 h and 48 h. Using these values, therapeutic concentration (fCmin>2 mg/L or >0.5 mg/L) was achieved for 48% or 64%, and 28% or 39% of patients, respectively. There was a high correlation between piperacillin and tazobactam concentrations at both time points (r = 0.97, *P* < 0.001 and r = 0.99, *P* < 0.001, respectively, Fig. S3, Supplemental material 2). Tazobactam concentrations at 24 h and 48 h had a Pearson correlation (r) of 0.46, *P* = 0.003.

A potentially neurotoxic antibiotic concentration did not occur in any patient on piperacillin-tazobactam (fCmin>160mg/L), and occurred in one patient (0/11 at 24 h, and 1/9 (11%) at 48 h) on meropenem (fCmin>64mg/L) (5). A potentially nephrotoxic concentration occurred in one additional patient on meropenem (fCmin>44.5 mg/L) at 24 h. Neither of the two patients with potential meropenem toxic concentrations had clinical evidence of nephrotoxicity (with either improvement or no change in urea and creatinine) or neurotoxicity (with either no change in sedation level or improvement in the level of consciousness and without seizures).

### Secondary outcomes: predictors of fCmin>MIC (EUCAST values) and time to resolution of infection

Predictors of fCmin>EUCAST MIC at 24 (*n* = 47) and 48 h (*n* = 38) are shown in Table 4 and Table 5. At 24 h, there was no independent predictor of fCmin>MIC (Table 4). At 48 h, ARC was the only independent predictor of therapeutic concentration with an OR of 0.05 (95% CI 0.00, 0.65; *P* = 0.022) (Table 5). Post-hoc analysis for the predictors of tazobactam fCmin>2 mg/L found that ARC was not a predictor at 24 h, but was a predictor at 48 h on multivariable logistic regression (controlling for nosocomial infection), with an OR of 0.06 (95% CI 0.00, 0.99; *P* = 0.049). Therefore, ARC was associated with a lower odds of piperacillin or meropenem fCmin>MIC and of tazobactam fCmin>2 mg/L at 48 h. Although there were only three patients on ECMO, this was not a predictor of fCmin>EUCAST MIC or >4× EUCAST MIC, attained at either time point in 3/4 measurements (comparison with non-ECMO patients, Fisher’s Exact *P* = 0.249) or 1/4 measurements (comparison with non-ECMO patients, Fisher’s Exact *P* = 0.469).

**TABLE 4:** Predictors of fCmin> ECOFF MIC at 24 h (n = 47)^*a*^

Variable	Univariate logistic regression		Multiple logistic regression	
	OR (95% CI)	*P*-value	OR (95% CI)	*P*-value
Sex (male)	0.92 (0.26, 3.2)	0.89		
ARC (*n* = 30)	0.26 (0.04, 1.56)	0.14	0.13 (0.01, 1.64)	0.115
ARC (≥75^th^)	0.31 (0.03, 3.07)	0.318		
ARDS	1.29 (0.39, 4.24)	0.680		
AKI	6.21 (0.59, 65.25)	0.128		
Septic shock	0.94 (0.27, 3.26)	0.925		
IMV	0.81 (0.19, 3.41)	0.777		
Weight (kg)	0.93 (0.86, 1.00)	0.060		
BSA (M^2^)	0.10 (0.01, 0.91)	0.041	0.01 (0.00, 3.31)	0.114
FA%	1.05 (0.98, 1.12)	0.160		
FA ≥ 10%	2.67 (0.74, 9.59)	0.133		
Nosocomial	1.42 (0.32, 6.41)	0.648		
Meropenem	4.55 (1.09, 18.98)	0.038	17.61 (0.63, 493.8)	0.092
PELOD-2	1.17 (0.88, 1.57)	0.279		
VIS	1.07 (0.96, 1.18)	0.242		
ECMO	3.87 (0.32, 46.18)	0.285		
Respiratory	1.16 (0.35, 3.84)	0.805		
Gram-negative bacilli on culture	0.83 (0.23, 3.03)	0.782		

^*a*^ AKI, acute kidney injury; ARC, augmented renal clearance; ARDS, acute respiratory distress syndrome; BSA, body surface area; ECMO, extracorporeal membrane oxygenation; ECOFF, European Committee on Antimicrobial Susceptibility Testing (EUCAST) epidemiological cutoff value, which indicated the highest MIC for *Pseudomonas aeruginosa *wild-type strains devoid of phenotypically detectable acquired resistance mechanisms; FA, fluid accumulation; IMV, invasive mechanical ventilation; PELOD, pediatric logistic organ dysfunction score; VIS, vasoactive inotrope score.

**TABLE 5:** Predictors of fCmin> ECOFF MIC at 48 h (n = 38)^*a*^

Variable	Univariate logistic regression		Multiple logistic regression	
	Odds ratio (95% CI)	*P*-value	Odds ratio (95% CI)	*P*-value
Sex (male)	0.90 (0.23, 3.59)	0.90		
ARC (*n* = 22)	0.06 (0.01, 0.50)	0.010	0.05 (0.00, 0.65)	0.022
ARC (≥75^th^)	0/5 vs. 8/17	0.115 (Fisher’s Exact)		
ARDS	0.58 (0.15 2.26)	0.430		
AKI	2/2 vs 11/36	0.111 (Fisher’s Exact)		
Septic shock	1.33 (0.33, 5.38)	0.691		
IMV	0.43 (0.09, 2.10)	0.297		
Weight (kg)	0.98 (0.94, 1.02)	0.286		
BSA (M^2^)	0.38 (0.08, 1.73)	0.209		
FA%	0.98 (0.91, 1.05)	0.554		
FA ≥ 10%	1.25 (0.31, 5.09)	0.755		
Nosocomial	4.67 (0.51, 42.92)	0.174		
Meropenem	13.42 (2.20, 82.00)	0.005	15.66 (0.73, 337.50)	0.079
PELOD-2	1.15 (0.83, 1.58)	0.408		
VIS	1.08 (0.95, 1.23)	0.256		
ECMO	1/1 vs. 12/37	0.342 (Fisher’s Exact)		
Respiratory	0.58 (0.15, 2.26)	0.430		
Gram-negative bacillus on culture	0.53 (0.12, 2.46)	0.420		

^*a*^ AKI, acute kidney injury; ARC, augmented renal clearance; ARDS, acute respiratory distress syndrome; BSA, body surface area; ECMO, extracorporeal membrane oxygenation; ECOFF, European Committee on Antimicrobial Susceptibility Testing (EUCAST) epidemiological cutoff value, which indicated the highest MIC for *Pseudomonas aeruginosa *wild-type strains devoid of phenotypically detectable acquired resistance mechanisms; FA, fluid accumulation; MIC, minimal inhibitory concentration; PELOD, pediatric logistic organ dysfunction score; VIS:,vasoactive inotrope score.

Predictors of time to resolution of infection in the subgroup of patients with an adjudicated confirmed infection (*n* = 32) are shown in Table 6. Respiratory site of infection was the only independent predictor, with an adjusted effect size of 72.96 (95% CI 24.95, 120.98; *P* = 0.004) h longer to resolution of signs of severe infection. Therapeutic antibiotic concentration was not associated with time to resolution of infection in univariate or multiple linear regressions.

**TABLE 6:** Predictors of time to resolution of signs of severe infection (n = 32)^*a*^

Variable	Univariate linear regression		Multiple linear regression	
	Effect size (95% CI)	*P*-value	Effect size (95% CI)	*P*-value
Sex (male)	30.49 (−24.79, 85.76)	0.269		
ARC (*n* = 18)	−8.41 (−53.33, 36.41)	0.697	−2.21 (−50.18, 45.75)	0.923
Any ARC ≥ 75^th^	20.34 (−29.65, 70.32)	0.403		
ARDS	20.64 (−29.22, 70.51)	0.405		
AKI	−2.90 (−108.16, 102.35)	0.955		
Septic shock	−27.33 (−78.57, 23.91)	0.285		
IMV	29.82 (−34.38, 94.01)	0.351		
Weight (kg)	−0.53 (−1.95, 0.89)	0.451		
BSA (M^2^)	−25.78 (−81.58, 30.02)	0.353		
FA%	0.41 (−2.12, 2.95)	0.741		
FA ≥ 10%	−5.28 (−58.77, 48.20)	0.841		
Nosocomial	−4.41 (−69.50, 60.69)	0.891		
Meropenem	−11.04 (−70.14, 48.06)	0.705	28.53 (−34.98, 92.05)	0.963
PELOD-2	2.73 (−8.86, 14.33)	0.634		
VIS	−0.46 (−4.66, 3.73)	0.823		
ECMO	32.23 (−72.37, 136.82)	0.534		
Respiratory	70.61 (26.87, 114.35)	0.002	72.96 (24.95, 120.98)	0.004^*b*^
Gram-negative bacillus on culture	−4.92 (−55.71, 45.86)	0.845		
fCmin > ECOFF MIC at either of 24 or 48 h (*n* = 31)^*b*^	−3.03 (−56.35, 50.30)	0.908	2.49 (−44.50, 49.48)	0.914^*b*^

^*a*^ AKI, acute kidney injury; ARC, augmented renal clearance; ARDS, acute respiratory distress syndrome; BSA: body surface area; ECMO, extracorporeal membrane oxygenation; ECOFF, European Committee on Antimicrobial Susceptibility Testing (EUCAST) epidemiological cutoff value, which indicated the highest MIC for *P. aeruginosa *wild-type strains devoid of phenotypically detectable acquired resistance mechanisms; FA, fluid accumulation; IMV, invasive mechanical ventilation; PELOD, pediatric logistic organ dysfunction syndrome score; VIS, vasoactive inotrope score.

^*b*^ Results for respiratory focus and therapeutic concentration are shown with only the two variables in the multiple regression (i.e., not forcing ARC or meropenem into the regressions, to avoid overfitting). When excluding cases with concomitant viral infection (n = 8), therapeutic concentration was not associated with time to resolution on univariate analysis, ES 17.91 (-32.56, 68.38) *P *= 0.469.

### Post-hoc outcome: predictors of ARC

Predictors of any ARC (*n* = 30, with 17 [57%] having ARC) are shown in Table S2. There were no predictors in the univariate or multiple logistic regression. None of the *n* = 3 patients with AKI had ARC, and none of the *n* = 2 not on invasive mechanical ventilation had ARC, but these numbers were too small to include in the multiple regressions and did not reach statistical significance in univariate analysis.

## DISCUSSION

The main findings of this prospective observational study were as follows: first, even using high daily doses of piperacillin-tazobactam or meropenem optimized for pharmacodynamics by administering every 6 h, subtherapeutic plasma concentrations of piperacillin and meropenem were common and persistent (when measured at both 24 and 48 h). This was the case both when using the target of fCmin>MIC or fCmin>4×MIC, and when using the measured MIC or the ECOFF MIC for *P. aeruginosa*. Using the ECOFF has been recommended, given that the clinical laboratory’s single measurement of MIC can be off by 1–2 dilutions, and therefore, using a high-end cutoff for wild-type bacterial organisms lacking resistance mechanisms is appropriate (4, 5, 18). Subtherapeutic concentrations of tazobactam were also common. Potentially toxic concentrations of either antibiotic were rare. Second, there were no independent, statistically significant clinical predictors found for a therapeutic antibiotic concentration (fCmin>MIC using ECOFF cutoff) at 24 h, and the only statistically significant independent predictor for a therapeutic concentration at 48 h was ARC (predicting a failure to achieve a therapeutic concentration). The OR of 0.05 with a wide 95% CI of 0.00, 0.65 (*P* = 0.022) warrants cautious interpretation. Third, ARC was common, persistent (when measured at both 24 and 48 h), and had no statistically significant independent predictor. Fourth, the time to resolution of severe symptoms and signs of sepsis were independently predicted by a respiratory focus of infection (associated with longer time to resolution), and not by a therapeutic antibiotic concentration.

These findings are compatible with previous research in which others have found suboptimal plasma antibiotic concentrations in critically ill children, including for piperacillin-tazobactam and meropenem (22–26). Paice et al. (*n* = 29 with severe sepsis, on meropenem mostly at lower doses of 20 mg/kg q8h) found a probability of target attainment (PTA) of 40%T > 4×MIC of 1 mg/L in 72% of patients in the first 24 h (22). Cies et al. (*n* = 14 on piperacillin-tazobactam, *n* = 16 on meropenem, with sepsis, using unspecified dosing regimens) found Cmin > MIC in 28% and 40% fT > 4×MIC in 11% of patients (23). Van Der Heggen et al. (*n* = 48 on relatively high doses of piperacillin-tazobactam 75 mg/kg q6h, *n* = 49 on meropenem 20–40 mg/kg q8h, in a critical care unit) found fCmin>MIC for 10% and 29% of patients, respectively, and an overall PTA of fCmin >4×MIC of 7.6% (24). Bradley et al. (*n* = 19 on relatively low doses of meropenem 20 mg/kg q8h, with septic shock) found fCmin>MIC 2 mg/L in 53% on day 1 of therapy (25). Finally, Maimongkol et al. (*n* = 72 on meropenem in a critical care unit) found that a relatively low dosing of 20 mg/kg q8h (*n* = 18) achieved fCmin>MIC of 1 or 2 mg/L in 33% or 28% of patients, and a higher dosing of 40 mg/kg/dose q8h by extended infusion (over 3 h, *n* = 54) achieved these targets in 71% and 51% of patients (26). It is difficult to compare these studies to our results because the dosing used was not uniformly high dose (or was unclear) or was not given every 6 h to optimize the time above a threshold target (22–26). Overall, these studies suggested that low doses of piperacillin-tazobactam or meropenem do not achieve reasonable pharmacodynamic targets, and even higher doses often fail when given in q8h regimens (including by extended infusion) (22–26). Our study used piperacillin-tazobactam 300 mg/kg/day q6h and meropenem 120 mg/kg/day q6h and found that fCmin>ECOFF MIC occurred in only 36% and 34% of patients at 24 and 48 h. To our knowledge, this was the first study to examine tazobactam concentrations in critically ill children and found these concentrations to be often subtherapeutic, perhaps expected, given the tight correlation between piperacillin and tazobactam plasma concentrations.

Others have also found that ARC is common in patients cared for in the PICU (6–8), is the main risk factor for subtherapeutic antibiotic concentrations, and is persistent or can develop anew (22, 24). Risk factors for ARC have included sepsis, febrile neutropenia, fluid therapy, vasoactive medication use, younger age, and higher CRP (8, 27). Each of these risk factors either could not be examined in the current study (e.g., almost all patients had sepsis and received fluid therapy), were not measured/recorded (e.g., CRP, febrile neutropenia), or were not confirmed (vasoactive medication, younger age). This may not be surprising, given that the risk factors for ARC were, as in our study, largely from single-center observational studies and therefore hypothesis-generating (8, 27). In addition, we included patients with normal renal function (e.g., only two (4%) with stage 3 AKI), and only 45% on vasoactive infusions, further limiting the detection of risk factors.

Our results are also compatible with population pharmacokinetic (PK) modeling studies in critically ill children (25, 46–54). These have often found heterogeneous Vd and clearance (CL) values between studies, sometimes had small sample sizes, did not directly measure CrCl (i.e., used eGFR equations), used scavenged opportunistic blood samples, and were not externally validated (25, 46–54). Nevertheless, the studies came to similar conclusions that standard intermittent infusion dosing of piperacillin or meropenem cannot achieve 90% PTA of pharmacodynamic Cmin targets. First, studies for piperacillin will be discussed. Butragueno-Laiseca et al. (*n* = 32) found that 100 mg/kg q6h (infused over 30 min) would achieve Cmin>MIC of 8 mg/L or 16 mg/L in 58% and 31% of patients, and 300 mg/kg/day continuous infusion would have 90% PTA of Cmin>MIC 16 mg/L (46). Girdwood et al. (*n* = 139) found that to achieve 90% PTA of Cmin>MIC 8 mg/L would usually require 88.9 mg/kg q4h (infused over 30 min), whereas with higher eGFR this would require dosing q2h; for 90% PTA of Cmin>16mg/L or 32 mg/L, either q2h dosing or continuous infusion of 356 mg/kg/day was required, and no regimen would achieve this for Cmin>64mg/L unless there was lower renal function (47). Ravix et al. (*n* = 104) found that only continuous infusion of 300 mg/kg/day would achieve 90% PTA of Cmin>MIC with normal renal function, and even prolonged infusions in those with ARC would not achieve this for MIC as low as 1 mg/L (48). De Cock et al. (*n* = 47) found that dosing 75 mg/kg q4h infused over 2 h, 100 mg/kg q4h infused over 1 h, or continuous infusion 300 mg/kg/day were “the minimal requirements to achieve the therapeutic targets for piperacillin 60% fT>MIC of 16 mg/L”; regimens of 75–100 mg/kg q6-8h infused over 30 min only had PTA between 5.9% and 34% (49). Similarly, Beranger et al. (*n* = 50) found standard dosing regimens had poor PTA and suggested 400 mg/kg/day in continuous or extended infusions (over 4 h q6h) (50). Second, studies for meropenem will be discussed. Morales Jr et al. (*n* = 48) found that achieving 90% PTA of fCmin>MIC 2 mg/L required continuous infusion, up to 120 mg/kg/day for fCmin>4× MIC 2 mg/L; standard dosing of 20–40 mg/kg q8h (infused over 30 min) was insufficient even to achieve 90% PTA of 40% fT>MIC 2 mg/L (51). This model was externally validated in *n* = 57 children receiving continuous infusion (52). Saito et al. (*n* = 34) found that standard dosing regimens provided insufficient exposures of Cmin>MIC 2 mg/L, and in patients with systemic inflammatory response syndrome, dosing needed to be 40–80 mg/kg q8h infused over 3 h (53). Cies et al. (*n* = 9) found that only 3–4 h prolonged infusion or continuous infusion achieved 90% PTA of 40% fT>MIC 4 mg/L, and 120–160 mg/kg/day as continuous infusion may be necessary to achieve 80% fT>MIC 4 mg/L (54). Finally, Bradley et al. (*n* = 19) found that in children with septic shock, 80 mg/kg q8h was required to achieve at least 90% fCmin>MIC 2 mg/L (25). Overall, these studies suggested dosing piperacillin-tazobactam at 300–400 mg/kg/day as continuous or extended infusions, and meropenem at 120–160 mg/kg/day as continuous or extended infusions.

In view of our finding of suboptimal target concentration attainment and the population PK data, continuous infusion of piperacillin-tazobactam and meropenem should be considered. In meta-analyses of randomized controlled trials (RCTs), continuous infusion of beta-lactam antibiotics (compared to intermittent dosing) reduced mortality and increased clinical cure in critically ill adults (2, 55). This is likely due to maintaining the Cmin above MIC continuously, instead of reducing below Cmin prior to the next dose with intermittent dosing. One systematic review and meta-analysis in children found that extended or continuous infusion was not associated with reduced mortality in five RCTs, but was associated with reduced mortality in eight observational studies and in two studies of critically ill children (56). Another systematic review and meta-analysis in children found lower all-cause mortality in both RCTs and observational studies, although this was statistically significant only in observational studies, and also reported better early microbiological eradication (57). The few studies in children, with low-quality evidence due to small sample sizes and heterogeneous, often non-critically-ill patients, prevent making clear conclusions (56, 57).

A potential solution to suboptimal target attainment may be therapeutic drug monitoring (TDM) for piperacillin and meropenem. Our data suggested that tazobactam TDM may not be necessary, given the very tight correlation between piperacillin and tazobactam levels. Some position papers and guidelines suggested real-time rapid-turnaround TDM in adult critically ill patients on beta-lactams (4, 5). Systematic review and meta-analyses of TDM for beta-lactams in critically ill adults found improved clinical cure and microbiological eradication and lower treatment failure; however, no difference in mortality was found (58, 59). Reasons for the lack of mortality difference included the low quality of studies (e.g., retrospective, small sample sizes, heterogeneous patient populations, including patients who may not have had confirmed infection, and those with low MIC bacteria, on variable beta-lactam and concomitant antibiotics), and variable dosing algorithms (58, 59). In addition, studies used different target goals, as the ideal target is unclear (59, 60). There is very limited evidence for TDM in critically ill children; one study using real-time TDM in *n* = 21 children on continuous infusion of a beta-lactam for documented gram-negative infections found that dose adjustments were recommended in 32.6% of measurements, and higher microbiological eradication and lower resistance development occurred with obtained Cmin>4× MIC (61, 62). A case can be made for TDM in critically ill children, given the low PTA for most potential target definitions, association of TDM-increased PTA with clinical cure and less development of antibiotic resistance, and the factors that uniquely affect critically ill patients (Table 1).

Most clinical studies in antibiotic development were undertaken in non-critically ill patients; however, the same dosing regimens are given in critically ill populations (60). The finding of subtherapeutic antibiotic concentrations may be explained by potential pharmacokinetic and pharmacodynamic considerations in critically ill children and is of particular concern in these critically ill children for several reasons (Table 1) (2–21). In the current study, the only predictor of time to resolution of signs of severe infection was pneumonia, likely because of a low tissue penetration ratio into epithelial lung tissue (e.g., 0.2–0.3 for meropenem) (13, 16), and possibly because of the subjectivity of adjudicating return of ventilator settings to a new baseline after a severe pneumonia. Nevertheless, we did not find that subtherapeutic antibiotic concentration was associated with time to resolution of signs of severe infection in the *n* = 32 patients where this could be examined. Other small pediatric studies have also failed to find an association with outcomes (22, 24–26). There are several potential reasons for these findings, including small sample sizes, often retrospective study designs, inclusion of patients who may not have had an infection at all or with an infection by a highly susceptible bacterial pathogen (with low MIC), concomitant antibiotics used, plasma concentrations being surrogates for tissue concentrations, lack of certainty on the optimal therapeutic target concentration, and lack of information on source control (58–60). In the current study, several of these limitations applied, and it was often difficult to determine whether there was a new bacterial infection being treated versus a deterioration due to a viral infection alone or worsening of an underlying disease condition.

This study has limitations. First, the sample size of *n* = 49 divided between two antibiotics, with not all participants having an antibiotic concentration measured at both time points, in two PICUs at one institution, limits the generalizability of the findings. Second, not all patients had a urinary catheter, and therefore, ARC could not be determined in all participants. Third, there was potential selection bias in the exclusion of patients with AKI based upon clinician judgment that dosing should be renally adjusted (rather than standardized criteria, which resulted in the inclusion of four patients with AKI, with one receiving RRT). However, this would be expected to improve target attainment (at least for the three patients with AKI not receiving RRT), making our estimates conservative. Fourth, we cannot be certain that participants had a bacterial infection; some had a viral illness (*n* = 8), many had pneumonia for which there was no gold-standard diagnostic test (e.g., VAP, *n* = 13), and many had an underlying illness that fluctuated and deterioration may not have been due to a new bacterial infection (e.g., cardiac *n* = 14 or noncardiac *n* = 18 surgery). This would not necessarily affect the attainment of therapeutic concentrations but would confound finding an association with time to symptom resolution. Fifth, we used a protein binding of 20% for piperacillin, which could be inaccurate in the critically ill. Sixth, there were almost certainly unmeasured confounders; the observational design can only detect associations and not make cause-and-effect conclusions. Finally, the target fCmin>MIC and fCmin>4×MIC were based upon *in vitro* bactericidal activity, and mostly upon consensus targets in adult critically ill patients (4, 5).

This study has several strengths. The study was prospective, using uniform antibiotic dosing optimized for beta-lactam pharmacodynamics, with many demographic, infection, and severity of illness (including ARC) variables clearly defined and recorded (blinded to antibiotic concentration measurements). Measurement of antibiotic concentrations was blinded to patient information. There were pre-specified primary and secondary outcomes, and respective statistical analysis plans. This is the first study to examine tazobactam concentrations in critically ill children.

### Conclusions

In critically ill children treated with piperacillin-tazobactam or meropenem at high doses divided q6h, measured Cmin often did not achieve pharmacodynamic targets, and rarely were toxic. The only independent predictor of subtherapeutic antibiotic concentrations was ARC (at 48 h), which occurred in approximately half of the patients. Although the time to resolution of signs of severe infection was not associated with a therapeutic antibiotic concentration, there were limitations to determining this outcome. Given that we found no robust predictors of ARC or subtherapeutic antibiotic concentrations, we suggest (i) dosing piperacillin-tazobactam at 300–400 mg/kg/day as continuous or extended infusions, and meropenem at 120–160 mg/kg/day as continuous or extended infusions; and (ii) that real-time rapid-turnaround TDM of piperacillin-tazobactam and meropenem be made available and used to optimize pharmacodynamic targets in critically ill children. Future research should examine the hypothesis that this will improve patient outcomes.

## Data Availability

The data used for this study are available on the Open Science Framework (OSF) at the following link: https://osf.io/kysp7/overview (Accessed March 23, 2026).

## SUPPLEMENTAL MATERIAL

Supplemental forms aac.01968-25-s0001.docx Case Report forms for the ATACC study.

Supplemental material aac.01968-25-s0002.docx Tables S1 and S2; Fig. S1 to S3; STROBE checklist.

ASM does not own the copyrights to Supplemental Material that may be linked to, or accessed through, an article. The authors have granted ASM a non-exclusive, world-wide license to publish the Supplemental Material files. Please contact the corresponding author directly for reuse.
