# Supplementary material for: Achieving therapeutic antibiotic levels during intermittent dosing of meropenem and piperacillin-tazobactam in critically ill children: the ATACC study
Source: Antimicrob Agents Chemother. 2026 May 28;70(7):e01968-25. doi: 10.1128/aac.01968-25 (PMC13321790; doi:10.1128/aac.01968-25)
Supplement: Supplemental forms — Case Report forms for the ATACC study. [file aac.01968-25-s0001.docx]

**Achieving Therapeutic Antibiotic Levels During Intermittent Dosing of Meropenem and Piperacillin-Tazobactam in Critically Ill Children: the ATACC study.**

**Authors:** Ari R Joffe MD^1^, Ashley Humber MD^2^, Angela Bates MD^1^, Jeffrey Lipman MD^3^, Steven Wallis PhD^3^, Jason Roberts BPharm (Hons), PhD^3-6^

**Journal**: Antimicrobial Agents and Chemotherapy

**Supplemental File 1**

**1. Case Report Form for the ATACC Study submitted to the health research ethics board December 11, 2020.**

**2. Case Report Form Manual for the ATACC Study submitted to the health research ethics board December 11, 2020.**

**ATACC Study**

**PATIENT CASE REPORT FORM**

*If found, please return to*:

Dr. A. Joffe or Dr. A. Bates

Stollery Children’s Hospital

Pediatric Intensive Care Unit and Pediatric Cardiac Intensive Care Unit

University of Alberta

**Form 1 – Inclusion/Exclusion Criteria:**

**1.1 Inclusion Criteria:**

Check “**yes**” for each inclusion criteria present.

**NOTE**: all inclusion criteria must be checked “yes” in order for the patient to be eligible for the study.

**1.1 INCLUSION CRITERIA**

# YES NO

___ ___ 1. Age 1 month to 17 years

___ ___ 2. Admitted to Stollery Children’s Hospital PICU or PCICU

___ ___ 3 Date is between January 2021 and December 2021

___ ___ 4. Prescribed meropenem or piperacillin-tazobactam intravenously

___ ___ 5. Anticipated duration of meropenem or piperacillin-tazobactam of at least 48 h

___ ___ 4. Informed consent: parent or legal guardian

**1.2 Exclusion Criteria:** Check “**no**” for each exclusion criteria NOT present.

**NOTE**: all exclusion criteria must be checked “no” in order for the patient to be eligible for the study.

**1.2 EXCLUSION CRITERIA**

**YES NO**

___ ___ 1. Acute or chronic kidney injury sufficient to require adjusted renal dosing of the

prescribed meropenem or piperacillin-tazobactam

___ ___ 2. Expected not to survive for 48 hours

___ ___ 3. Refusal of informed consent by the parent or legal guardian.

***FORM 2. DEMOGRAPHICS AND RISK FACTORS***

**2-1. Age at initiation of study antibiotic: *|_____| months***

**2-2. Date of initiation of study antibiotic: *|__|__| |__|__|__| |__|__|__|__|***

***D D / M M M / Y E A R***

**2-3. Gender at birth: *|__| Male |__| Female***

**2-4. Weight: *|__|__|.|__|kg***

**2-5. Height *|__|__|__|cm***

**2-6. Body Surface Area (BSA) *|__|.__|__|m^2^***

**2-7. Admission Diagnostic Category: *Please list all diagnoses made by the treating team***

**Sepsis:**  ***|__| Yes |__| No***

**severe sepsis; septic shock**

**Neurologic (medical): *|__| Yes |__| No***

**altered level of consciousness; seizures; stroke; meningitis; encephalitis**

**other ­­­­­­­­­­____________________**

**Respiratory: *|__| Yes |__| No***

**bronchiolitis; bacterial pneumonia (community, hospital, or ventilator associated);**

**asthma; aspiration; Cystic Fibrosis exacerbation**

**other _____________________________**

**Cardiac (medical): *|__| Yes |__| No***

**myocarditis; Cardiomyopathy; Dysrhythmia; Endocarditis**

**other ___________________________**

**Abdominal: *|__| Yes |__| No***

**bowel obstruction; bowel perforation; ulcer; pancreatitis**

**other __________________________**

**Renal: *|__| Yes |__| No***

**acute kidney injury, chronic kidney injury**

**other ________________________________**

**Surgical, Cardiac: *|__| Yes |__| No***

**single ventricle, congenital bi-ventricular, acquired valvular disease,**

**other _________________________________**

**Surgical, non-cardiac: *|__| Yes |__| No***

**neurosurgery, chest surgery, abdominal surgery, musculoskeletal surgery**

**other ___________________________________**

**Surgical, transplant: *|__| Yes |__| No***

**liver, heart, kidney**

**FORM 3: Infection Data**

**3-1. Infection type**

**Community acquired *|__| Yes |__| No***

**Nosocomial *|__| Yes |__| No***

**3-2. Infection confirmation**

**Confirmed *|__| Yes |__| No***

**Not confirmed** ***|__| Yes |__| No***

**3-3. Site of infection suspected**

**CNS *|__| Yes |__| No***

**meningitis, brain abscess**

**other ______________________**

**Respiratory *|__| Yes |__| No***

**pneumonia, empyema, lung abscess**

**other ______________________**

**Cardiac *|__| Yes |__| No***

**endocarditis, mediastinitis**

**other ______________________**

**Abdominal *|__| Yes |__| No***

**bowel perforation (includes appendicitis), pancreatic infection, post-operative abdominal surgery other ____________________________________**

**Urinary tract *|__| Yes |__| No***

**UTI**

**Other ___________________________**

**Musculoskeletal *|__| Yes |__| No***

**fasciitis, cellulitis, osteomyelitis (this includes spinal rod infections)**

**other ___________________________**

**Device related *|__| Yes |__| No***

**broviak, IVAD, CVL, pacemaker**

**other __________________________**

**Unknown *|__| Yes |__| No***

**3-4. Suspected causative organism (in confirmed infections only)**

**Gram Negative Bacillus** ***|__| Yes |__| No***

E. coli ***|__|__|.|__| mg/L***

Klebsiella sp. ***|__|__|.|__| mg/L***

Enterobacter sp. ***|__|__|.|__| mg/L***

Citrobacter sp. ***|__|__|.|__| mg/L***

Pseudomonas aeruginosa ***|__|__|.|__| mg/L***

Serratia sp. ***|__|__|.|__| mg/L***

Acinetobacter sp. ***|__|__|.|__| mg/L***

Anaerobes: Bacteroides sp. ***|__|__|.|__| mg/L***

Other _____________________________ ***|__|__|.|__| mg/L***

**Gram Positive Coccus *|__| Yes |__| No***

S. aureus ***|__|__|.|__| mg/L***

Streptococcus pneumonia ***|__|__|.|__| mg/L***

viridans group Streptococcus ***|__|__|.|__| mg/L***

Group A or C Streptococcus ***|__|__|.|__| mg/L***

Other _______________________________ ***|__|__|.|__| mg/L***

**Gram Negative Cocci or Coccobacilli *|__| Yes |__| No***

Haemophilus sp. ***|__|__|.|__| mg/L***

Moraxella sp. ***|__|__|.|__| mg/L***

Neisseria meningitidis ***|__|__|.|__| mg/L***

Neisseria gonorrhea ***|__|__|.|__| mg/L***

Other ____________________________ ***|__|__|.|__| mg/L***

**No positive culture obtained *|__| Yes |__| No***

**3-5. Creatinine clearance:**

**At ~24 hours At ~48 hours**

Urine sample obtained ***|__| Yes |__| No |__| Yes |__| No***

Serum creatinine: ***|__|__| umol/L |__|__| umol/L***

Urine creatinine: ***|__|__| umol/L |__|__| umol/L***

Urine volume: ***|__|******__|__| ml |__|__|__| ml***

Minutes of urine collection ***|__|__|__| minutes |__|__|__| minutes***

Creatinine clearance ***|__|__|__| ml/min |__|__|__| ml/min/1.73m^2^***

ARC-1 ***|__| Yes |__| No*** ***|__| Yes |__| No***

ARC-2 ***|__| Yes |__| No |__| Yes |__| No***

**3-6. Antibiotic level:**

**At ~24 hours At ~48 hours**

**Meropenem *or |__|__|.|__| mg/L*** ***|__|__|.|__| mg/L***

**Piperacillin-tazobactam *|__|__|.|__| mg/L |__|__|.|__| mg/L***

**Form 4: Severity of Illness**

**4-1: PELOD-2 Score** from 24 hours before to 4 hours after antibiotic prescription

**Total PELOD-2 Score: *|__|__| points***

**4-2. Inotrope score** ***|__|__|__|.|__|* *points***

**4-3. ARDS Category *|__| Yes |__| No***

Mild ARDS ***|__| Yes |__| No***

Moderate ARDS ***|__| Yes |__| No***

Severe ARDS ***|__| Yes |__| No***

ARDS not categorized ***|__| Yes |__| No***

CPAP or BIPAP ***|__| Yes |__| No***

Cyanotic heart disease ***|__| Yes |__| No***

Chronic lung disease normally ventilated ***|__| Yes |__| No***

**4-4. AKI *|__| Yes |__| No***

Stage 1  ***|__| Yes |__| No***

Stage 2 ***|__| Yes |__| No***

Stage 3  ***|__| Yes |__| No***

Stage 2 or 3 (severe) ***|__| Yes |__| No***

**4-5. Sepsis *|__| Yes |__| No***

Severe Sepsis ***|__| Yes |__| No***

Septic Shock ***|__| Yes |__| No***

Hypotension ***|__| Yes |__| No***

Impaired perfusion ***|__| Yes |__| No***

Vasoactive medication ***|__| Yes |__| No***

**4-6. Ventilation *|__| Yes |__| No***

Invasive ***|__| Yes |__| No***

Non-invasive ***|__| Yes |__| No***

**4-7. % Fluid Overload since ICU admission + or – (circle which)**

***|__|__|.|__| %***

Within 4 hr at 24h level at 48h level

**4-8. CRRT use** ***|__| Yes |__| No* *|__| Yes |__| No |__| Yes |__| No***

CVVH ***|__| Yes |__| No* *|__| Yes |__| No |__| Yes |__| No***

CVVHD ***|__| Yes |__| No* *|__| Yes |__| No |__| Yes |__| No*** CVVHDF ***|__| Yes |__| No* *|__| Yes |__| No |__| Yes |__| No***

**Other RRT in use *|__| Yes |__| No* *|__| Yes |__| No |__| Yes |__| No***

IHD (intermittent hemodialysis) ***|__| Yes |__| No* *|__| Yes |__| No |__| Yes |__| No***

Peritoneal Dialysis ***|__| Yes |__| No* *|__| Yes |__| No |__| Yes |__| No***

Within 4 hr at 24h level at 48h level

**4-9. ECLS use *|__| Yes |__| No* *|__| Yes |__| No |__| Yes |__| No***

VV ECMO ***|__| Yes |__| No* *|__| Yes |__| No |__| Yes |__| No***

VA ECMO ***|__| Yes |__| No* *|__| Yes |__| No |__| Yes |__| No***

VAD ***|__| Yes |__| No* *|__| Yes |__| No |__| Yes |__| No***

***FORM 5. Outcomes:***

**5-1. Primary outcomes**

**At ~24 hours At ~48 hours**

**Antibiotic given by intermittent q6h dosing** ***|__| Yes |__| No |__| Yes |__|No***

**Antibiotic given by continuous infusion dosing**  ***|__| Yes |__| No |__| Yes |__|No***

MIC known ***|__| Yes |__| No |__| Yes |__|No***

MIC value ***|__|__|.|__| mg/L*** ***|__|__|.|__| mg/L***

fT>MIC of 100% (level is above MIC) ***|__| Yes |__| No |__| Yes |__|No***

fT>4X MIC of 100% (level is above 4X MIC) ***|__| Yes |__| No |__| Yes |__|No***

fT>2mg/L for meropenem, or >20mg/L for pip-tazo ***|__| Yes |__| No |__| Yes |__|No***

fT>8mg/L for meropenem, or >80mg/L for pip-tazo ***|__| Yes |__| No |__| Yes |__|No***

Toxicity: level >16mg/L for meropenem, ***|__| Yes |__| No |__| Yes |__|No***

or >160 mg/L for pip-tazo

**5-2. Secondary outcomes**

Confirmed infection: ***|__| Yes |__| No***

Time to resolution of signs of severe infection ***|__|__|__| hours***

Time to treating clinical team judging the patient as ‘resolved infection’ ***|__|__|.|__| days***

Infection was the reason for needing ICU admission: ***|__| Yes |__| No***

Time to ‘ready for discharge’ as determined by the treating clinical team ***|__|__|.|__| days***

**5-3. Mortality**

Alive or discharged from hospital at 30d from antibiotic prescription  ***|__| Yes |__| No***

Death by 30d from antibiotic prescription ***|__| Yes |__| No***

***FORM 6. WITHDRAWAL FROM THE STUDY***

| **Withdrawal from study** | ***\|__\| yes \|__\| no*** |
| --- | --- |
| ***If yes****, check the appropriate reason* | |
| 21-1 Parents asked to withdraw the child from the study:  (*Justification if given:____________________________________* ) | ***\|__\| yes \|__\| no*** |
| 21-2 Physician asked to withdraw the child from the study:  (*Justification:________________________________* ) | ***\|__\| yes \|__\| no*** |
| 21-3 Other cause of withdrawal:  (*Specify:*_______________________________________ ) | ***\|__\| yes \|__\| no*** |
| 21-4 Date of withdrawal | *\|__\|__\| \|__\|__\|__\| \|__\|__ \|__\|__\|*  *DD M M M Y Y Y Y* |

***FORM 7. SIGN OFF SHEET***

Case Report Form to be signed off when the data has been checked as accurate and complete.

**Research Assistant:** __________________________________  **Date:** _____________________

**Study Investigator:** __________________________________ **Date:** ______________________

**ATACC Study**

**CASE REPORT FORM MANUAL**

*If found, please return to*:

Dr. A. Joffe or Dr. A. Bates

Stollery Children’s Hospital

Pediatric Intensive Care Unit and Pediatric Cardiac Intensive Care Unit

University of Alberta

**Case Report Forms: Procedure Manual Notes**

**General Instructions**

· At the top of each page, enter the **Patient’s Study** **Number**

· Enter dates in the format dd / mm / yyyy (i.e. October 22, 1998 is 22 / Oct / 1998)

· Enter times according to the 24 hour clock in the format HH:MM (i.e. 4 pm is entered 16:00)

· Do not write in shaded areas

· If data is not applicable, not known, illegible, or incorrect, enter N/A. There should be no blank spaces.

· Use only **black ink**

**Identifying information will be kept separately in a locked cabinet in the locked research office.**

**Form 1. Inclusion/Exclusion Criteria:**

All inclusion must be ‘yes’, and all exclusion must be ‘no’

Form 2. Demographics:

**Date of initiation of meropenem or piperacillin-tazobactam**: calendar date

**Age at initiation of meropenem or piperacillin-tazobactam**: Please record the child’s age in months.

Record one month if the baby is aged 30 to 59 days.

Record two months if the baby is 60-89 days, etc.

**Sex at birth**: Check either male or female

**Weight**: in kg

**Height**: in cm

**BSA**: in M^2^

**Admission Diagnostic Category:** Record the diagnoses made by the treating team **on admission** to the ICU.

Categories: please check the category as yes/no, and then circle the appropriate sub-diagnosis within that category. If ‘other’ is the best answer, please write in the description of the diagnosis.

**Form 3. Infection data:**

This is for the infection being treated, that is, having the meropenem or piperacillin-tazobactam being prescribed to treat.

**Infection type:**

-Community acquired: present on admission or developed within 48 hours of admission to hospital

-Nosocomial: detected over 48 hours after admission to hospital

**Infection confirmation:**

-confirmed by the clinical team: decision to treat for at least 5 days with antibiotics

-not confirmed by the clinical team: decision to stop antibiotics for that possible infection before 5 full days of antibiotic administration for that possible infection

**Suspected site of infection:** Record the suspected site determined by the treating team in the ICU.

Categories: please check the category as yes/no, and then circle the appropriate sub-site. If ‘other’ is the best answer, please write in the description of that infection site.

If unsure, please talk to Ari (in PICU), Angela (in PCICU), or Ashley for clarification.

**Suspected causative organism: found on culture for confirmed infections**, from the suspected site listed above (or blood and thought to originate from the suspected site listed above), and being treated with meropenem or piperacillin-tazobactam.

Answer yes/no for the category of organism, and circle the organism in that category below. Also write the MIC provided by the microbiology laboratory for that causative organism(s).

There can be multiple pathogens for some infections. Do not record the following potential pathogens (and their MIC) that are not sensitive to nor treated with meropenem or piperacillin-tazobactam:

Gram Positive Cocci: Coagulase Negative Staphylococcus sp., MRSA

Gram Negative Bacilli: Stenotrophomonas sp., Burkholderia sp.

Other: Legionella sp., Mycoplasma sp., Listeria sp., Bacillus sp., Corynebacterium sp.

Non-bacterial: fungal, viral, parasitic

**Creatinine clearance: determined in those with a foley catheter (or suprapubic catheter), at 24 and 48 hours on antibiotics.**

Serum creatinine: Screat in umol/L. Use the value closest to the urine collection at 24h and 48h. If not re-measured, use the value closest to the day of urine collection.

Urine creatinine: Ucreat in umol/L

Urine volume: Uv in millilitres

Minutes of urine collection: in minutes

Creatinine clearance:

(Uv)(Ucreat)(1.73) / (BSA)(Screat)(minutes of urine collection)

ARC-1: defined as creatinine clearance above 90^th^ percentile from the table below

ARC-2: defined as above the 75^th^ percentile of this study, and will be determined later.

Creatinine clearance norms:

**Age (years) 90^th^ percentile CrCl (ml/min/1.73m^2^)**

**_____________________________________­­­­­­­­­­­­­­­­­­­­**

0.10 56.3

0.20 70.9

0.30 80.9

0.40 87.3

0.50 93.6

0.60 99.8

0.70 100.9

0.80 107.3

0.90 110.0

1.00 111.8

1.10 112.7

1.20 115.5

1.30 118.2

1.40 119.5

1.50 120.5

1.60 121.8

1.70 123.6

1.80 123.6

1.90 124.5

2.00 125.5

2+ 131.2

References:

Piepsz A, Tondeur M, Ham H. Revisiting normal ^51^Cr-ethylenediaminetetraacetic acid clearance values in children. Eur J Nucl Med Mol Imagint 2006;33:1477-1482.

38. Schwarz GJ, Work DF. Measurement and estimation of GFR in children and adolescents. Clin J Am Soc Nephrol 2009;4:1832-1843.

**Plasma antibiotic level:**

Determined from CAMPA data at end of study.

***Form 4. Severity of illness measures***

Record the following **as the worst values within 4 hours of the time that the meropenem or piperacillin-tazobactam were prescribed**.

**4-1. PELOD-2 Score**: use the sheet provided. Use the **worst values in the 24 hours leading up to, and up to 4 hours after the time of prescribing** the antibiotic. Circle the appropriate score for each organ, and give the total score.

If a criterion is not measured, it is assumed to be within the normal range for scoring purposes. If it is measured more than once in 24 hours, the most severe value is used in calculating the score.

- Heart rate: do not assess during crying or iatrogenic agitation.
- Systolic blood pressure: do not assess during crying or iatrogenic agitation.
- PaO_2_/FiO_2_ ratio calculation: e.g., PaO_2_ = 89 and FiO_2_ = 45% = 0.45

Therefore, PaO_2_/FiO_2_ = 89/0.45 = 198.

Mechanical ventilation: the use of mask ventilation is not considered as mechanical ventilation.

Glasgow coma score: use the lowest value. If the patient is sedated, paralyzed or intubated, record the estimated Glasgow coma score before these events and/or use the best documented notation.

- Pupillary reactions: non reactive pupils must be > 3 mm. Do not assess after iatrogenic pupillary dilatation

Reference: Reference: Leteurtre S, Duhamel A, Salleron J, Grandbastien B, Lacroix J, Leclerc F. PELOD-2: an update of the Pediatric Logistic Organ Dysfunction Score. Crit Care Med 2013;41(7):1761-1763.

**4-2.** **Inotrope Score**: calculate as follows. The worst value **within 4 hours of the time of the prescription** of the study antibiotic.

Reference:

McIntosh AM, Tong S, Deakyne SJ, Davidson JA, Scott HF. Validation of the vasoactive-inotrope score in pediatric sepsis. Pediatr Crit Care Med 2017;18:750-757.

**4-3. ARDS Category:** determine from the table, the worst category **within 4 hours of the time of the prescription** of the study antibiotic, and categorize as:

No ARDS: not meeting any of the criteria for ARDS

If ARDS: categorize as one of the following:

Mild ARDS

Moderate ARDS

Severe ARDS

ARDS not categorized: this can be due to any one of the following -

CPAP or BIPAP: currently in use

Cyanotic heart disease: patient with a BT shunt, Hybrid procedure, Norwood, Sano, Glenn, or Fontan

Chronic lung disease that normally receives invasive ventilation via endotracheal tube or tracheostomy, even if periods up to 6 hours off are allowed. If periods off of 6 hours or more are used, use criteria not applicable to chronic lung disease (i.e., categorize ARDS if possible).

Reference:

The Pediatric Acute Lung Injury Consensus Conference Group. Pediatric acute respiratory distress syndrome: consensus recommendations from the pediatric acute lung injury consensus conference. Pediatr Crit Care Med 2015;16:428-439.

**4-4. AKI Category**: determine as follows, the worst category **within 4 hours of the time of prescription** of the antibiotic.

When the two criteria (Scr and urine output) result in different stages, the **greater severity stage** will be used.

**Baseline SCr**: is the lowest SCr in the 3 months preceding admission. When baseline SCr was unavailable, the average SCr norms for age and sex used in Alberta clinical laboratories will be used. If a patient has only one SCr value measured during PICU admission, the criteria of absolute SCr increase of greater than or equal to 26.5 mmol/L will not be applied.

If not meeting any of the criteria: mark as AKI “no”

We define **severe AKI as KDIGO stage 2 and 3**.

Kidney Disease Improving Global Outcome (KDIGO) AKI Definition Criteria for AKI

| **Stage** | **Serum Creatinine** | **Urine Output** |
| --- | --- | --- |
| 1 | 1.5–1.9 times baseline  OR  Increase of ≥ 0.3 mg/dl (≥ 26.5 mmol/l) | < 0.5 ml/kg/h for 6–12 hours |
| 2 | 2.0–2.9 times baseline | < 0.5 ml/kg/h for ≥ 12 hours |
| 3 | 3.0 times baseline  OR  Increase of ≥ 4.0 mg/dl (≥ 353.6 mmol/l)  OR  Initiation of renal replacement therapy  OR  In patients < 18 years, decrease in eGFR to < 35 ml/min per 1.73 m2 | < 0.3 ml/kg/h for ≥ 24 hours  OR  Anuria for ≥ 12 hours |

Reference: Selewski DT, Cornell TT, Heung M, et al. Validation of the KDIGO acute kidney injury criteria in a pediatric critical care population. Intensive Care Med 2014; 40:1481–1488.

The Average (Range) SCr Norms for Age and Sex in Alberta Clinical Laboratories (in μmol/L)

| **Age** | **Female** | **Male** |
| --- | --- | --- |
| 0-23 months | 30 (10-40) | 30 (10-40) |
| 2-5 years | 32 (20-45) | 32 (20-45) |
| 6-12 years | 48 (20-75) | 48 (20-75) |
| 13-14 years | 63 (30-95) | 63 (30-95) |
| 15-150 years | 70 (40-100) | 85 (50-120) |

**4-5. Sepsis Category**: determine as follows, as the worst category **within 4 hours of the prescription** of the study antibiotic.

If not meeting any of the criteria below, mark as sepsis “no”

**“Severe sepsis”**: Sepsis associated organ dysfunction - severe infection leading to cardiovascular and/or noncardiovascular organ dysfunction.

-we will use this to replace the old definition of Severe Sepsis as infection with at least two SIRS criteria and either cardiovascular dysfunction, ARDS, or at least two noncardiovascular organ system dysfunction

-organ dysfunction will be a score of at least 1 on the PELOD-2 score

**Septic Shock**: severe infection leading to cardiovascular dysfunction, including **hypotension, need for treatment with a vasoactive medication, or impaired perfusion**.

-anyone with septic shock also fulfils the severe sepsis definition

-record which criteria the patient meets: hypotension, vasoactive medication, and/or impaired perfusion

-vasoactive medication includes any of: dopamine, dobutamine, epinephrine, norepinephrine, vasopressin, phenylephrine, milrinone

-**only include vasoactive medication criterion if**: the patient has this medication started within 4 hours of the antibiotic prescription, or if already on the vasoactive medication, had an increase of more than 10% in the dose within 4 hours of the antibiotic prescription

Impaired perfusion: defined as any t**wo of the following**:

Unexplained base deficit -5.0 mEq/L or more negative

Arterial lactate > 2mmol/L

Urine output <0.5 ml/kg/hr

Prolonged capillary refill time of > 5 secs

Mottled cool extremities

New decreased or altered mental status

Diminished palpable pulses

References:

Goldstein B, Giroir B, Randolph A, and the members of the International Consensus Conference on Pediatric Sepsis. International pediatric sepsis consensus conference: definitions for sepsis and organ dysfunction in pediatrics. Pediatr Crit Care Med 2005;6:2-8.

Davis AL, Carcillo JA, Rajesh A, Deymann AJ, Lin JC, Nguyen T, et al. American College of Critical Care Medicine clinical practice parameters for hemodynamic support for pediatric and neonatal septic shock. Crit Care Med 2017;45(6):1061-1093.

(ii) Hypotension: defined as SBP *or* MAP below the 5^th^ percentile for age according to the table below.

Reference:

Joffe AR. Importance of hypotension and its definition after cardiac arrest. JAMA Pediatr 2018;172(2):120-122.

**4-6. Ventilation**: record as one of the following, **within 4 hours of the prescription** of the antibiotic:

If not meeting either of the below, mark as ventilation “no”. Oxygen by nasal prongs or face mask that does not meet the criteria above is considered ‘no’.

Invasive: ventilation via an endotracheal tube or tracheostomy

Non-invasive: by face-mask that is either full face or nasal, either CPAP or BIPAP

High-flow nasal cannula: at a flow rate at least 1ml/kg/minute)

**4-7. % Fluid Overload since ICU admission**

-if admitted for more than 5 full days, use the % fluid overload over the past 72 hours

-as a % of admission to PICU weight = cumulative fluid balance in liters / weight in kg

-if the number is a negative number, record it as negative and the %

**4-8. CRRT use**: **within 4 hours of the prescription** of study antibiotic, use of any of CVVH, CVVHD, CVVHDF

Other dialysis: includes IHD (intermittent hemodialysis), or Peritoneal Dialysis

Also record if any of these were in use at the time of the 24h and 48h antibiotic level

**4-9. ECLS use**: **within 4 hours of the prescription** of study antibiotic, use of any of VV ECMO, VA ECMO, or VAD. If both VV and VA, record only VA as the mode of ECLS.

-also record if this was in use at the time of the 24h and 48h antibiotic level

**Form 5. Outcomes**

***Primary outcome****:* Incidence of therapeutic antibiotic drug levels achieved in intermittent dosing and continuous infusions of meropenem and piperacillin-tazobactam. A therapeutic drug level is defined as %fT>k X MIC of 100%. Here, k indicates that the desired level is related to a multiple of the MIC of the organism detected or suspected. We will use the following methods to determine the definition of a therapeutic drug level:

| **Definition of therapeutic level** | **Meropenem** | **Piperacillin-tazobactam** |
| --- | --- | --- |
| MIC available | fT>MIC of 100% | fT>MIC of 100%  This is: level >MIC/0.8 |
|  | fT > 4 X MIC of 100% | fT > 4X MIC of 100%  This is: level >4X MIC/0.8 |
| MIC using the ECOFF | fT > MIC using 2mg/L of 100% | fT > MIC using 20 mg/L of 100% |
|  | fT > 4 X MIC which is >8 mg/L of 100% | fT > 4 X MIC which is >80 mg/L of 100% |
| Definition of toxicity | Total level > 16 mg/L | Total level > 160 mg/L |

-If MIC is known, record the highest MIC value for known organism(s) causing the infection from section 3-4 above, and use it to determine if fT is above that MIC or above 4X that MIC. To correct for protein binding for piperacillin-tazobactam, the level needs to be above MIC/0.8 or 4XMIC/0.8 respectively. Using ECOFF, the table above already corrects for protein binding.

***Secondary outcomes:***

In those with confirmed infection:

a) Time to resolution of signs of severe infection: criteria for this are – back to baseline (at least 12 hours prior to antibiotic prescription), **in hours from when antibiotic was prescribed**

-inotrope score

-ventilation settings (PEEP, delta P, and rate)

-level of consciousness (by GCS), and

-temperature (afebrile)

b) Time to treating clinical team judging the patient as ‘resolved infection’: in 0.5 days units.

-ask team for time in 12 hour increments from time of antibiotic prescription

3. If infection was the reason for needing ICU admission: in 0.5 days units.

a) time to ‘ready for discharge’ as determined by the treating clinical team

-ask team for time in 12 hour increments from time of antibiotic prescription

**Mortality**: by 30 days from date of study antibiotic prescription.

**Form 6—Withdrawal from the study:**

**WITHDRAWAL FROM THE STUDY:** Check "yes" if the patient has been withdrawn from the study within 30 days after inclusion, and check the appropriate reason.

**Form 7 – Sign off sheet:**

# Sign Off Sheet

**This sheet must be completed. By signing this page the parties state that the forms have been reviewed and are deemed complete and accurate**.
