# Supplementary material for: Achieving therapeutic antibiotic levels during intermittent dosing of meropenem and piperacillin-tazobactam in critically ill children: the ATACC study
Source: Antimicrob Agents Chemother. 2026 May 28;70(7):e01968-25. doi: 10.1128/aac.01968-25 (PMC13321790; doi:10.1128/aac.01968-25)
Supplement: Supplemental material — Tables S1 and S2; Fig. S1 to S3; STROBE checklist. [file aac.01968-25-s0002.docx]

**Achieving Therapeutic Antibiotic Levels During Intermittent Dosing of Meropenem and Piperacillin-Tazobactam in Critically Ill Children: the ATACC study.**

**Authors:** Ari R Joffe MD^1^, Ashley Humber MD^2^, Angela Bates MD^1^, Jeffrey Lipman MD^3^, Steven Wallis PhD^3^, Jason Roberts BPharm (hons), PhD^3-6^

**Journal**: Antimicrobial Agents and Chemotherapy

**Supplementary Material File 2**

**eTable 1.** More detailed descriptive data for the 49 critically ill children included in the study.

**eTable 2.** Predictors of any Augmented Renal Clearance: creatinine clearance was measured in n=30, with 17 (57%) having augmented renal clearance.

**eFigure 1**. Meropenem (n=11 and n=9) and Piperacillin (n=36 and n=29) minimum free antibiotic concentrations (fCmin in mg/L) at 24-hours and 48-hours.

**eFigure 2.** Correlation between minimum free antibiotic concentrations (fCmin in mg/L) at 24-hours and 48-hours in n=38 patients.

**eFigure 3.** Correlation between piperacillin and tazobactam minimum free concentration (fCmin, in mg/L) at 24 hours and 48 hours.

**Strobe Checklist**

**eTable 1.** More detailed descriptive data for the 49 critically ill children included in the study.

| **Variable** | **Result (n=49)** | |
| --- | --- | --- |
| **Demographics** | | |
| Age (months) | 58 (72); 17 [6, 116] | |
| Sex (female) | 17 (35%) | |
| Weight (kg) | 18.4 (20.1); 10.4 [5.7, 20.0] | |
| Height (cm) | 122 (216) | |
| Body Surface Area (M^2^) | 0.66 (0.52); 0.47 [0.30, 0.92] | |
| **Admission diagnostic category** | | |
| Sepsis  Severe sepsis  Septic shock | 5  2  3 | |
| Neurological  Altered level of consciousness  Seizures  Stroke  Meningitis  Encephalitis  Other | 6  2  2  1  0  0  4 | |
| Respiratory  Bronchiolitis  Bacterial pneumonia  Asthma  Aspiration  Cystic Fibrosis exacerbation  Other | 11  5  4  0  1  0  5 | |
| Cardiac  Myocarditis  Dysrhythmia  Endocarditis  Other | 2  0  0  0  2 | |
| Abdominal  Bowel obstruction  Bowel perforation  Ulcer  Pancreatitis  Other | 1  0  0  0  0  1 | |
| Renal  Acute Kidney Injury  Chronic Kidney Injury  Other | 1  0  0  1 | |
| Surgical Cardiac  Single ventricle  Bi-Ventricular  Acquired valvular disease  Other | 14  2  8  1  4 | |
| Surgical Noncardiac  Neurosurgery  Chest surgery  Abdominal surgery  Musculoskeletal surgery  Other | 12  2  0  3  2  6 | |
| Surgical Transplant  Liver  Heart  Kidney  Other | 6  4  2  0  0 | |
| **Infection information** | | |
| Infection type  Community acquired  Nosocomial | 10 (20%)  39 (80%) | |
| Infection confirmation  Treated ≥5 days with antibiotics  Treated <5 days of antibiotics  Confirmed site of infection | 33 (67%)  16 (33%)  34 (69%) | |
| Sepsis  Severe sepsis  Septic shock  Hypotension  Vasoactive infusion  Impaired perfusion | 47 (96%)  47 (96%)  18 (37%)  11 (22%)  14 (29%)  5 (10%) | |
| Site of infection  Respiratory (pneumonia)  Ventilator associated  Hospital acquired  Community acquired  Cardiac (mediastinitis)  Abdominal  Bowel perforation  Intra-abdominal abscess  Other (peritonitis, NEC,  ischemic bowel)  Urinary Tract infection  CLABSI (CVL, PICC, IVAD)  Not confirmed or prophylaxis | 34 (69%)  20  13  2  5  1  6  1  2  3  7  3  15 (31%) | |
| **Infecting organism** | | |
| Gram Negative Bacilli  *E. coli*  *Klebsiella sp.*  *Enterobacter sp.*  *Citrobacter sp.*  *Pseudomonas aeruginosa*  *Serratia sp.*  *Acinetobacter sp*.  Anaerobic: *Bacteroides sp.*  Other (*Pluribacter gergoviae*) | 16 (33%)  1  6  5  0  6  1  0  0  1 | |
| Gram Positive Cocci  *S. aureus*  *Streptococcus pneumonia*  Viridans group streptococcus  Group A or C *Streptococcus*  Other (*Enteroccocus sp*. 2,  *Cutibacterium acnes* 1) | 9 (18%)  5  1  0  0  3 | |
| Gram negative cocci or coccobacilli  *Haemophilus sp.*  *Moraxella sp.*  *Neisseria meningitidis*  *Neisseria gonorrhea*  Other | 4 (8%)  1  3  0  0  0 | |
| No positive culture | 25 (51%) | |
| **Clinical condition at time of antibiotic prescription** | |  |
| **Time** | **24 hours** | **48 hours** |
| Creatinine clearance  Serum creatinine (µmol/L)  Urine creatinine (umol/L)  Urine volume (ml)  Minutes of urine collection (min)  Creatinine clearance (ml/min/1.73M^2^) | n=30  37 (26)  2893 (3168)  200 (196)  266 (135)  110 (81); 107 [57, 155] | n=22  34 (26)  3555 (5170)  177 (178)  245 (27)  118 (65); 132 [60, 169] |
| Augmented renal clearance (>90^th^ percentile for age)^a^ | 13/30 (43%) | 14/22 (64%) |
| On RRT when level drawn | 1 | 0 |
| On ECMO when level drawn | 3 | 3 |
| PELOD-2 score  Neurologic  Cardiovascular  Renal  Respiratory  Hematologic | 5.1 (2.2); 5 [3, 6.5]  16 (33%)  34 (69%)  10 (20%)  40 (82%)  12 (24%) | |
| Vasoactive Inotrope Score  On Vasoactive(s)  Dopamine  Dobutamine  Epinephrine  Milrinone  Vasopressin  Norepinephrine | 3.6 (5.7); 0 [0, 5.5]  22 (45%)  -  -  13 (27%)  5 (10%)  -  10 (20%) | |
| Acute Respiratory Distress Syndrome  Mild  Moderate  Severe  Not categorized: mask CPAP or BIPAP | 24 (49%)  15 (31%)  6 (12%)  2 (4%)  1 (2%) | |
| Acute Kidney Injury within 4 hours of prescription of antibiotic  Stage 1  Stage 2  Stage 3  Urine output >0.5 ml/kg/hr | 4 (8%)  1  1  2  46 (94%) | |
| Ventilated  Invasive ventilation  Non-Invasive ventilation | 45 (92%)  38 (78%)  7 (14%) | |
| Fluid accumulation since admission (%)  Fluid accumulation ≥10% | 7.3 (10.2); 5.2 [0.9, 12.8]  15 (31%) | |
| **Outcomes** | | |
| Time to resolution of signs of severe infection (n=32) | 84.3 (68.6); 65.0 [36.9, 111.0]; range 10-324 | |
| Mortality within 30 days | 2 (4%) | |

Continuous variables presented as mean (standard deviation); median [interquartile range]. Categorical variables presented as number (percentage).
BIPAP: bilevel positive airway pressure; CLABSI: Central Line Associated Bloodstream Infection; CPAP: continuous positive airway pressure; CVL: central venous line; ECMO: extracorporeal membrane oxygenation; IVAD: implanted venous access device; NEC: necrotizing enterocolitis; PELOD-2: pediatric logistic organ dysfunction score; PICC: peripherally inserted central line; RRT: renal replacement therapy.

a. ARC measured at both time points (n=22): yes to no 0 (0%); no to yes 4 (18%); both yes 10 (45%); both no 8 (36%).

**eTable 2.** Predictors of any Augmented Renal Clearance: creatinine clearance was measured in n=30, with 17 (57%) having augmented renal clearance.

| **Variable** | **Univariate logistic regression** | | **Multiple logistic regression** | |
| --- | --- | --- | --- | --- |
|  | **OR (95% CI)** | **p-value** | **OR (95% CI)** | **p-value** |
| Sex (Male) | 1.50 (0.33, 6.92) | 0.603 |  |  |
| ARDS | 2.06 (0.46, 9.30) | 0.349 |  |  |
| AKI | 0/3 vs 17/27 | 0.070 (Fisher’s Exact) | NA |  |
| Septic shock | 1.67 (0.39, 7.15) | 0.492 |  |  |
| IMV | 17/28 vs 0/2 | 0.179 (Fisher’s Exact) | NA |  |
| Weight (kg) | 1.01 (0.98, 1.05) | 0.418 |  |  |
| BSA (M^2^) | 1.42 (0.38, 5.40) | 0.603 |  |  |
| FA% | 1.00 (0.91, 1.10) | 0.990 |  |  |
| FA ≥10% | 1.02 (0.21, 4.98) | 0.978 |  |  |
| Nosocomial | 0.55 (0.11, 2.81) | 0.472 |  |  |
| Meropenem | 0.44 (0.06, 3.16) | 0.417 | 0.38 (0.05, 2.95) | 0.353 |
| PELOD | 0.78 (0.52, 1.17) | 0.235 |  |  |
| VIS | 0.95 (0.84, 1.06) | 0.348 |  |  |
| ECMO | 0.34 (0.03, 4.27) | 0.406 |  |  |
| Respiratory | 0.34 (0.08, 1.52) | 0.159 | 0.32 (0.07, 1.47) | 0.141 |
| GNB on culture | 0.94 (0.19, 4.52) | 0.936 |  |  |

AKI: acute kidney injury; ARDS: acute respiratory distress syndrome; BSA: body surface area; ECMO: extracorporeal membrane oxygenation; FA: fluid accumulation; GNB: gram negative bacilli; IMV: invasive mechanical ventilation; PELOD: pediatric logistic organ dysfunction score 2; VIS: vasoactive inotrope score.

None with AKI (n=3) had augmented renal clearance (ARC )or ARC ≥75^th^ percentile, and therefore this was not included in the multiple regressions. None without invasive ventilation had ARC, and therefore this was not included in the multiple regressions. The number with AKI (n=3) and without IMV (n=2) having ARC measured is too few to understand their association with ARC.

**eFigure 1**. Meropenem (n=11 and n=9) and Piperacillin (n=36 and n=29) minimum free antibiotic concentrations (fCmin in mg/L) at 24-hours and 48-hours.


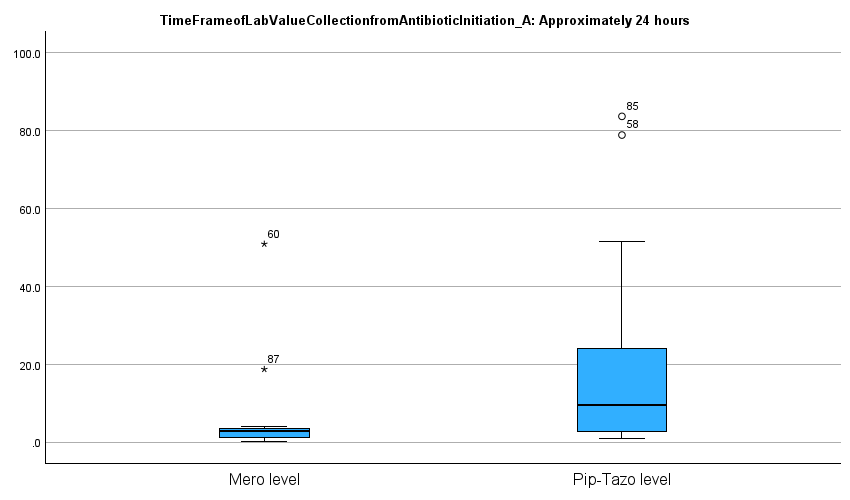


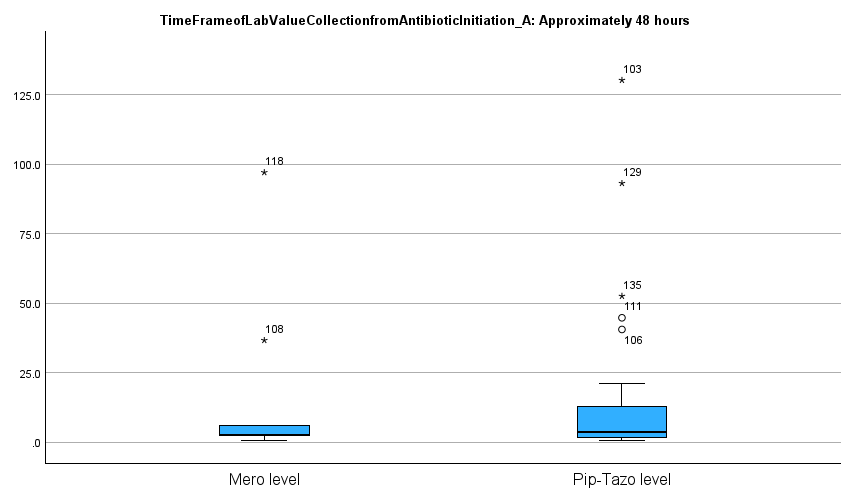


**eFigure 2.** Correlation between minimum free antibiotic concentrations (fCmin in mg/L) at 24-hours and 48-hours in n=38 patients.


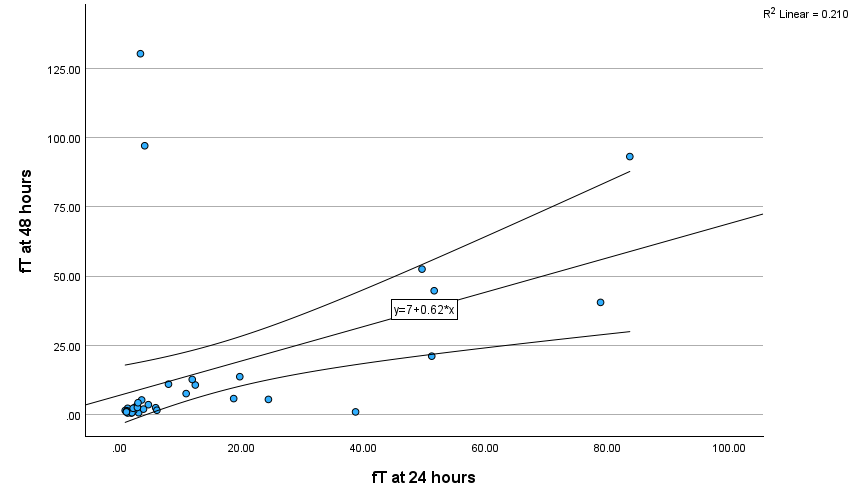


**e-Figure 3.** Correlation between piperacillin and tazobactam minimum free concentration (fCmin, in mg/L) at 24 hours and 48 hours.


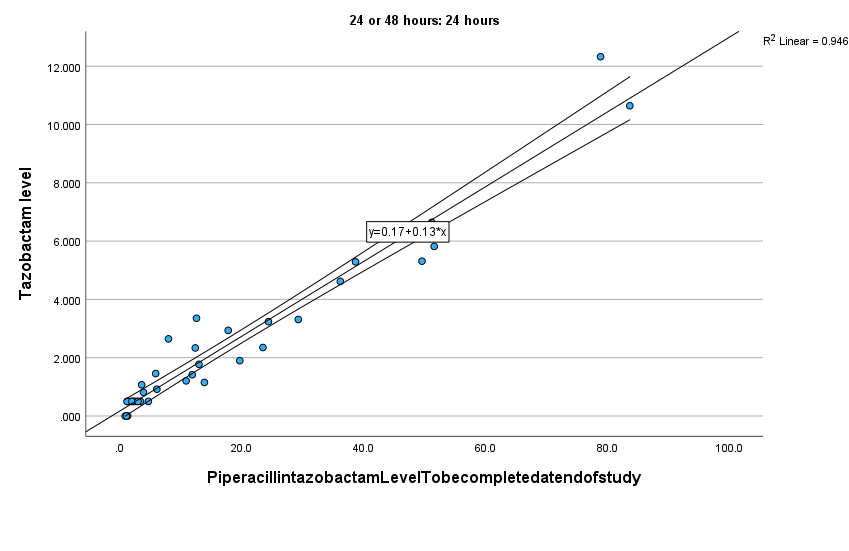


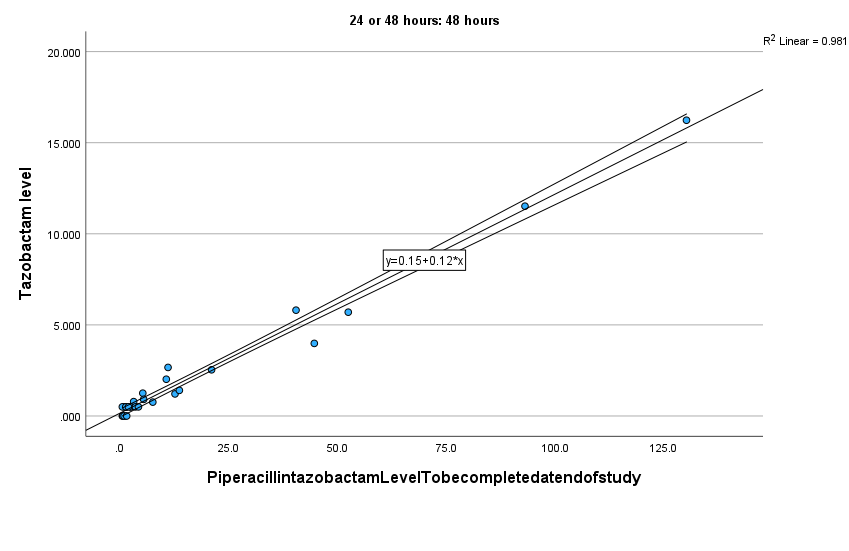


STROBE Statement—Checklist of items that should be included in reports of ***cohort studies***

|  | Item No | Recommendation | Page No |
| --- | --- | --- | --- |
| **Title and abstract** | 1 | (*a*) Indicate the study’s design with a commonly used term in the title or the abstract | 1 |
|  |  | (*b*) Provide in the abstract an informative and balanced summary of what was done and what was found | 2 |
| Introduction | | | |
| Background/rationale | 2 | Explain the scientific background and rationale for the investigation being reported | 3,4 |
| Objectives | 3 | State specific objectives, including any prespecified hypotheses | 4 |
| Methods | | | |
| Study design | 4 | Present key elements of study design early in the paper | 5 |
| Setting | 5 | Describe the setting, locations, and relevant dates, including periods of recruitment, exposure, follow-up, and data collection | 5  Figure 1 |
| Participants | 6 | (*a*) Give the eligibility criteria, and the sources and methods of selection of participants. Describe methods of follow-up | 5,6 |
|  |  | (*b*) For matched studies, give matching criteria and number of exposed and unexposed | N/A |
| Variables | 7 | Clearly define all outcomes, exposures, predictors, potential confounders, and effect modifiers. Give diagnostic criteria, if applicable | 5-7 |
| Data sources/ measurement | 8* | For each variable of interest, give sources of data and details of methods of assessment (measurement). Describe comparability of assessment methods if there is more than one group | 5-7 |
| Bias | 9 | Describe any efforts to address potential sources of bias | 7  Supplemental Material File 1 |
| Study size | 10 | Explain how the study size was arrived at | 8 |
| Quantitative variables | 11 | Explain how quantitative variables were handled in the analyses. If applicable, describe which groupings were chosen and why | 7,8 |
| Statistical methods | 12 | (*a*) Describe all statistical methods, including those used to control for confounding | 7,8 |
|  |  | (*b*) Describe any methods used to examine subgroups and interactions |  |
|  |  | (*c*) Explain how missing data were addressed |  |
|  |  | (*d*) If applicable, explain how loss to follow-up was addressed |  |
|  |  | (*e*) Describe any sensitivity analyses |  |
| Results | | |  |
| Participants | 13* | (a) Report numbers of individuals at each stage of study—eg numbers potentially eligible, examined for eligibility, confirmed eligible, included in the study, completing follow-up, and analysed | 9, Figure 1 |
|  |  | (b) Give reasons for non-participation at each stage |  |
|  |  | (c) Consider use of a flow diagram |  |
| Descriptive data | 14* | (a) Give characteristics of study participants (eg demographic, clinical, social) and information on exposures and potential confounders | 9,10  Table 1  eTable 1 |
|  |  | (b) Indicate number of participants with missing data for each variable of interest |  |
|  |  | (c) Summarise follow-up time (eg, average and total amount) |  |
| Outcome data | 15* | Report numbers of outcome events or summary measures over time | Table 2 |

| Main results | 16 | (*a*) Give unadjusted estimates and, if applicable, confounder-adjusted estimates and their precision (eg, 95% confidence interval). Make clear which confounders were adjusted for and why they were included | Tables 3 to 5  eTable 2 |
| --- | --- | --- | --- |
|  |  | (*b*) Report category boundaries when continuous variables were categorized |  |
|  |  | (*c*) If relevant, consider translating estimates of relative risk into absolute risk for a meaningful time period |  |
| Other analyses | 17 | Report other analyses done—eg analyses of subgroups and interactions, and sensitivity analyses | 11  eFigures 1-3 |
| Discussion | | | |
| Key results | 18 | Summarise key results with reference to study objectives | 11,12 |
| Limitations | 19 | Discuss limitations of the study, taking into account sources of potential bias or imprecision. Discuss both direction and magnitude of any potential bias | 13,14 |
| Interpretation | 20 | Give a cautious overall interpretation of results considering objectives, limitations, multiplicity of analyses, results from similar studies, and other relevant evidence | 14,15 |
| Generalisability | 21 | Discuss the generalisability (external validity) of the study results | 13 |
| Other information | | | |
| Funding | 22 | Give the source of funding and the role of the funders for the present study and, if applicable, for the original study on which the present article is based | 16 |

*Give information separately for exposed and unexposed groups.

**Note:** An Explanation and Elaboration article discusses each checklist item and gives methodological background and published examples of transparent reporting. The STROBE checklist is best used in conjunction with this article (freely available on the Web sites of PLoS Medicine at http://www.plosmedicine.org/, Annals of Internal Medicine at http://www.annals.org/, and Epidemiology at http://www.epidem.com/). Information on the STROBE Initiative is available at http://www.strobe-statement.org.
